# Detection and identification of plant leaf diseases using YOLOv4

**DOI:** 10.3389/fpls.2024.1355941

**Published:** 2024-04-22

**Authors:** Eman Abdullah Aldakheel, Mohammed Zakariah, Amira H. Alabdalall

**Affiliations:** ^1^ Department of Computer Sciences, College of Computer and Information Sciences, Princess Nourah bint Abdulrahman University, Riyadh, Saudi Arabia; ^2^ Department of Computer Science, College of Computer and Information Science, King Saud University, Riyadh, Saudi Arabia; ^3^ Department of Biology, College of Science, Imam Abdulrahman Bin Faisal University, Dammam, Saudi Arabia

**Keywords:** plant leaf disease, leaf disease detection, object detection, deep-learning, YOLO v4, darknet

## Abstract

Detecting plant leaf diseases accurately and promptly is essential for reducing economic consequences and maximizing crop yield. However, farmers’ dependence on conventional manual techniques presents a difficulty in accurately pinpointing particular diseases. This research investigates the utilization of the YOLOv4 algorithm for detecting and identifying plant leaf diseases. This study uses the comprehensive Plant Village Dataset, which includes over fifty thousand photos of healthy and diseased plant leaves from fourteen different species, to develop advanced disease prediction systems in agriculture. Data augmentation techniques including histogram equalization and horizontal flip were used to improve the dataset and strengthen the model’s resilience. A comprehensive assessment of the YOLOv4 algorithm was conducted, which involved comparing its performance with established target identification methods including Densenet, Alexanet, and neural networks. When YOLOv4 was used on the Plant Village dataset, it achieved an impressive accuracy of 99.99%. The evaluation criteria, including accuracy, precision, recall, and f1-score, consistently showed high performance with a value of 0.99, confirming the effectiveness of the proposed methodology. This study’s results demonstrate substantial advancements in plant disease detection and underscore the capabilities of YOLOv4 as a sophisticated tool for accurate disease prediction. These developments have significant significance for everyone involved in agriculture, researchers, and farmers, providing improved capacities for disease control and crop protection.

## 1 Introduction

Plant diseases present a crucial obstacle to the growth of agriculture in every country, resulting in significant yearly financial losses (Mitra, 2021). Plant disease detection has developed into a substantial area of study in pattern recognition and contemporary agricultural development due to developments in machine learning technology (Roy and Bhaduri, 2021; Albattah et al., 2022; Sanida et al., 2023). Early plant disease identification approaches used a support vector machine (SVM) (Rahman et al., 2023; Thangavel et al., 2023), artificial neural network (ANN) (Attallah, 2023), and SVM method for disease diagnosis under segmented plant disease spots. These techniques are used to manually isolate the affected area of an image, after which the K-means clustering method is implemented (Javidan et al., 2023).

With the advancement of AI technology, agricultural detection based on AI is now widely utilized for tasks including predicting crop production, processing weed identification, and finding plant diseases (Albattah et al., 2022). Moreover, the process of machine learning-based disease detection involves several steps. Firstly, the dataset undergoes preprocessing to ensure its suitability for analysis. Following this, feature extraction algorithms are employed to identify and extract relevant features from regions of interest in the images, specifically focusing on disease-affected areas of plant leaves. Subsequently, the extracted feature information is transmitted to the classifier, where model parameters are derived. Finally, the system accepts the identified categories of diseases, along with their respective severity levels, integrating this crucial information into the output for further analysis or action. Moreover, leveraging image recognition through machine learning methodologies holds significant promise for enhancing the generalization ability of models. Specifically, in the domain of detecting and identifying plant leaf diseases using YOLOv4, the term “model generalization ability” pertains to the model’s proficiency in accurately identifying and categorizing diseases in plant leaves across a broad spectrum of scenarios, including instances not encountered during training. This capability enables the model to effectively discern subtle patterns and characteristics indicative of various leaf diseases, thereby contributing to more reliable and robust disease detection systems (Singh et al., 2017). When there are fewer classes, it is easier to distinguish between their characteristics. Moreover, the categories can only be identified within minimal visual settings. Researching a fast, end-to-end plant disease detection system is vital since it will be necessary to meet the demands of large-scale planting.

Further, early identification and management of plant diseases is an essential part of crop harvesting, since it helps to minimize development problems and lowers the need for pesticides. As a result, the environmental damage brought on by pesticide use is reduced, supporting sustainable agricultural practices (Perveen et al., 2023). Many ML techniques have been used for plant and disease categorization and detection.Where such approaches, however, perform less well and more slowly when detecting diseases in real time (Peng and Wang, 2022), likely due to the problematic image preprocessing and feature extraction stages. The fact that classic ML methods are unsuitable for real-world detection scenarios with complex backgrounds and non-uniform surfaces is another major disadvantage of these methods. With several applications, deep learning has lately achieved a substantial breakthrough in this area of computer vision (Wang et al., 2021).

Additionally, it has been used for picture segmentation, crop recognition, and automated agriculture technology, including the classification of crops and fruits (Dhinesh et al., 2019). Models based on convolutional neural networks (CNNs) have gained popularity due to their improved accuracy in object detection (Sangeetha et al., 2022). CNNs can save time on preprocessing because they automatically extract features from the input images. The ability to identify crop diseases has made great strides in recent years (Dhinesh et al., 2019; Sangeetha et al., 2022). There are now two distinct kinds of CNN-based object detectors: those with a single detection stage and those with two. One of the most popular two-stage detectors is the region convolution neural network (RCNN), which consists of the fast/faster RCNN and the mask-RCNN (Sangeetha et al., 2022). These models have had significant effects on automated agriculture management (Chen et al., 2022), crop and fruit detection (Eunice et al., 2022), and yield and growth assessment (Hassan et al., 2021). Although these models cannot recognize high-resolution images in real-time, faster R-CNN, consisting of region proposal networks (RPN) (Chen and Wu, 2023) and classification networks, considerably decrease detection time. By combining target categorization and localization into a regression problem, the recently suggested You Only Look Once (YOLO) (Soeb et al., 2023) method simplifies the problem. Due to its lack of RPN, YOLO employs regression to directly locate targets in the image, significantly improving its detection speed. High precision, accuracy, and detection speed are hallmarks of modern object detection technologies like YOLOv4 (Xinming and Tang, 2023), which can perform several real-time object recognition applications.

This work (Chuanlei et al., 2017) concentrates on rust and scab detection in apple leaves, which are susceptible to two dangerous and prevalent fungal infections. Real-time early disease identification of apple leaves is challenging due to factors such as the fine-grained multi-scale distribution, the similarity of colour texture between illnesses and background, the diversity of diseases’ morphology, and the occurrence of many diseases on the same leaf. A significant gap exists between the current model and real-time illness diagnosis on mobile computing devices since existing disease detection algorithms pay off accuracy for real-time detection speed (Arathi and Dulhare, 2023).

Plant leaves have many different features, such as differences in size, shape, color, and growth conditions, but effectively identifying and categorizing diseases is still quite difficult. Brightness fluctuations that occur during the process of capturing leaf image aggravate this issue and challenge detection strategies. This paper presents a novel approach that leverages the YOLOv4 architecture to achieve remarkable accuracy on the Plant Village dataset. It is shown that the used methodology is robust against a range of input sample distortions, including noise, blurring, rotation, contrast changes, lighting adjustments, color inconsistencies, and brightness swings. The outcomes highlight how well the recommended approach works to improve plant leaf disease identification and detection.

**Figure 1:**
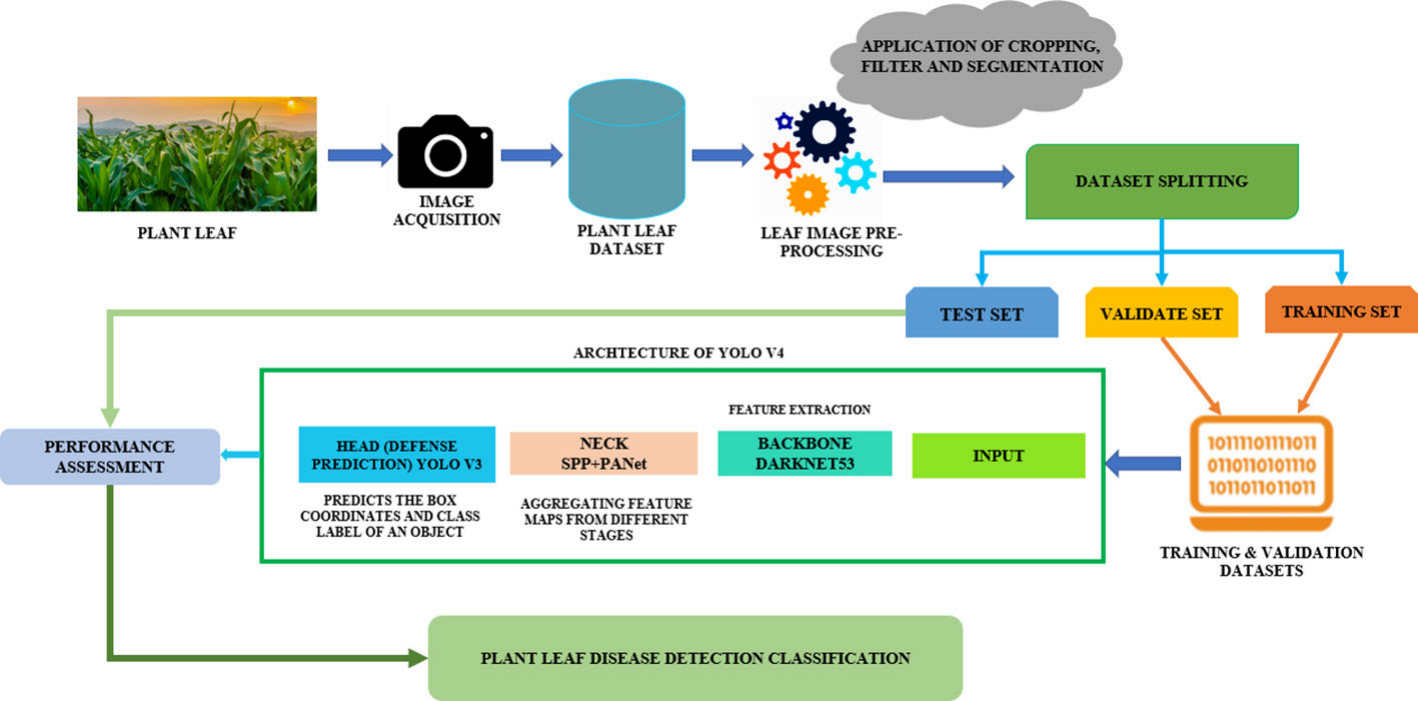
Implementing the Yolov4 disease detection and identification framework in plant leaves.

To create a framework for plant disease detection and classification, the following processes must be completed: data collection, model training, and multiple-class categorization of plant leaf disease. **Figure 1** provides an overview of the proposed framework. Leaf disease items can be found and identified with the Yolov4 method.

The following are the primary contributions of this research paper:

- This study applies YOLOv4, a cutting-edge object detection framework, to plant pathology. The work improves plant leaf disease identification accuracy and efficiency by seamlessly incorporating YOLOv4, providing a fresh method to address agricultural concerns.
- The study achieves high precision in recognizing and classifying various plant leaf diseases through thorough image annotation and data preprocessing. The suggested methodology’s robustness provides accurate disease diagnosis, assisting farmers and researchers in timely intervention and crop management.
- The study uses the widely accepted Plant Village dataset, which helps validate and benchmark the proposed model. This dataset selection improves the generalizability of the findings, allowing for more excellent applications in plant pathology research.
- The research provides valuable insights into the practical implementation of the YOLOv4 architecture for detecting plant leaf disease. The precise methodology can help researchers and practitioners implement and adapt this technology in real-world agricultural contexts.

The fundamental structure of this paper is as follows: An overview of the topic is given in the first part. The literature on diagnosing leaf diseases is reviewed in the section 2. The dataset structure and the evaluation criteria for the experiments are presented in the 3 section. The methodology portion is covered in section 4, and a comparison of the findings is covered in section 5. The discussion portion is covered under Section 6. Finally, a summary concludes in section 7, where the paper is closed up.

## 2 Literature review

In the context of precision agriculture, there is a strong need for sophisticated techniques that improve detection efficiency because plant diseases pose significant risks to crop health and the field (Attallah, 2023; Rahman et al., 2023; Thangavel et al., 2023). In computer vision for plant disease identification, deep learning—specifically, the YOLOv4 architecture—has proven to be a potent tool. Deep learning holds great potential to overcome obstacles associated with various illness kinds and environmental variables (Javidan et al., 2023). Previous research has demonstrated the drawbacks of standard approaches. The release of YOLOv4 improves object identification speed and accuracy, which makes it an excellent option for applications in plant pathology (Singh et al., 2017). An in-depth analysis of the current research environment is provided by this literature review, which sets the stage for a later investigation into the use of YOLOv4 in identifying and detecting plant leaf diseases (Perveen et al., 2023).

By addressing the fundamental restrictions that are present in the approaches that are currently in use, Peng et al (Peng and Wang, 2022). have made great achievements in the advancement of plant disease identification. Predefined recognition categories and the significant demand for image annotation are two examples of these restrictions that are particularly difficult to overcome. The authors have methodically separated the PlantVillage dataset into two sub-datasets, which they have referred to as PlantVillage-A and PlantVillage-B. This allowed them to use the PlantVillage dataset as a solid foundation for training and evaluation. Remarkable accuracy rates of 98% for YOLOv5 and 97.84% for ResNet were achieved by their proposed strategy, which indicates a significant change from the standard approaches that have been historically utilized. Not only does this achievement serve as a testimonial to the effectiveness of the technique in minimizing the inadequacies of previous methodologies, but it also serves to enhance the precision and adaptability of leaf disease identification. A full re-evaluation of recognition categories and an improved method to picture annotation are both components of the strategy that has been presented.

Further, Chen et al. (2022) introduced an improved model based on the YOLOv5 network to precisely identify diseases in the difficult conditions of various natural habitats, significantly contributing to plant disease recognition and using the PlantVillage dataset—especially the rubber tree disease database with 2375 images—the suggested model targets anthracnose and powdery mildew. The model achieves an impressive 86.5% accuracy rate, highlighting its usefulness in improving methods for identifying plant diseases.

Moreover, Eunice et al. (2022) address the critical imperative of early plant disease diagnosis for optimizing food production and reducing economic losses. Leveraging the advancements in deep learning, the study introduces a robust leaf disease detection model for agricultural applications. The focus lies on utilizing CNN-based pre-trained models, specifically DenseNet-121 and VGG-16, with meticulous hyperparameter fine-tuning. The PlantVillage dataset, comprising 54,305 samples, serves as the comprehensive foundation partitioned for training, validation, and testing. Impressively, the proposed model achieves an exceptional accuracy of 99.81%, underscoring its effectiveness in enhancing plant disease identification accuracy and its potential for substantial impact on agricultural practices and crop management strategies (Hassan et al., 2021; Chen and Wu, 2023; Soeb et al., 2023; Xinming and Tang, 2023).

Likewise, Chuanlei et al. (2017) contributed to the critical domain of apple leaf disease identification within computer vision, addressing the challenge of adequate representation of diseased leaf images. Leveraging the PlantVillage dataset, comprising ninety images, the study employs a methodology centred on a Support Vector Machine (SVM) classifier. Impressively, the proposed model achieves an accuracy surpassing 95% on the PlantVillage dataset, showcasing the efficacy of the developed apple leaf disease recognition method (Arathi and Dulhare, 2023; Bin Naeem et al., 2023; Xu et al., 2023). This work holds substantial promise for advancing computer vision applications in precision agriculture and plant health monitoring (Vengaiah and Konda, 2023; Terentev et al., 2023; Liu et al., 2023).

Further, Kaur et al. (2022) contributed significantly to the imperative task of plant disease identification for sustainable agriculture. Acknowledging the challenges of manual monitoring, the research focuses on grapevines, targeting four prevalent diseases: Leaf blight, Black rot, stable, and Black measles (Taheri-Garavand et al., 2021; Sharma et al., 2023). With the overarching need for automated disease characterization in agriculture, the study utilizes the PlantVillage dataset, emphasizing grapevine health with 2115 images. The proposed Hybrid Convolutional Neural Network emerges as a robust solution, achieving a remarkable accuracy of 98.7%. This work stands at the forefront of precision agriculture, offering a comprehensive approach to automatic and accurate leaf disease recognition in grape plants (Mustafa et al., 2020).

Moreover, Jasim et al (Jasim and Al-Tuwaijari, 2020). contributed to precision agriculture with their research using Image Processing and Deep Learning Techniques. The study leverages the comprehensive Plant Village dataset comprising 20,636 images to address the critical task of detecting and classifying plant leaf diseases. Further, it Employs deep-learning techniques, particularly Convolutional Neural Networks (CNN), where the research advances the state-of-the-art in disease identification (Liu and Wang, 2021). Using CNN allowed for the classification of 15 distinct classes, encompassing various diseases such as bacteria and fungi, along with a category for healthy leaves. The proposed model attained an accuracy of 90.029%, underscoring its efficacy in the automated detection and classification of plant leaf diseases, thereby contributing significantly to advancing precision agriculture methodologies.

To sum up, this study has advanced plant pathology significantly. The study effectively solves the crucial need for precise and effective plant leaf disease detection and classification using the YOLOv4 architecture. The research shows that picture annotation, data preparation, and model training, that is used to diagnose various diseases through creative integration and rigorous methods reliably. High precision is achieved with the help of the YOLOv4 model, demonstrating the model’s potential for practical application in agriculture. This approach facilitates prompt interventions and sustainable crop management practices, contributing to the evolving field of precision agriculture.

**Table 1** contains a list of previous references along with plant types, techniques, limitations and outcomes.

**Table 1:** List of past references with datasets, methodology, and results.

References	Datasets	Methodology	Limitations	Results
(Peng and Wang, 2022)	**- **The Plant Village dataset is used. **- **It consists of around thirty-eight thousand and thirty-five images for Plant Village-A dataset and sixteen thousand two-hundred and seventy images for Plant Village-B.	YOLO V5, ResNet-50	The research paper’s limitation is its potential for generalizability, as the performance of the proposed picture retrieval system may vary across different datasets and environments.	The resNet model has an accuracy of 97.84% for the Plant Village dataset.
(Wang et al., 2021)	PlantVillage dataset consists of images of 14 plants with around 3000 images.	Squeeze-and-excitation SSD (Se_SSD), deep block SSD (DB_SSD).	The research needs to improve in generalizability, as the algorithm’s performance may vary across diverse plant diseases or datasets beyond the specific conditions of the PlantVillage dataset.	This model has an accuracy of 92.20% using the same Plant Village dataset.
(Chen et al., 2022)	The Plant Village dataset consists of around two thousand three-hundred seventy-five images.	YOLOv5 network model	The research may need to be revised in assessing the model’s robustness across diverse plant diseases and datasets beyond the rubber tree disease database, impacting its generalizability.	The accuracy of this model is 86.5%.
(Eunice et al., 2022)	The Plant Village dataset consists of fifty thousand three hundred and five images.	DenseNet-121, VGG-16	The research’s limitations may hinder its ability to extend its deep learning-based leaf disease detection model across diverse crops and environmental conditions.	This model has an accuracy of 99.81%
(Chuanlei et al., 2017)	The Plant Village dataset consists of 90 images	SVM Classifier	The research may need to be revised in generalizability, as the proposed genetic algorithm and correlation-based feature selection method may not seamlessly extend to diverse plant diseases or datasets beyond apple leaf diseases.	This model has an accuracy of 94.22%
(Kaur et al., 2022)	The Plant Village dataset consists of one hundred 1,500 healthy images.	Hybrid Convolutional Neural Network	The research’s focus on grapevines may limit its generalizability, and the hybrid convolutional neural network may need to be more easily adapted to detect a broader range of plant illnesses.	This model has an accuracy of 98.7%
(Jasim and Al-Tuwaijari, 2020)	The Plant Village dataset consists of twenty thousand six-hundred thirty-six images.	Deep-Learning Techniques, CNN	The paper’s limitations include potential issues extending the proposed system to other plant species and diseases beyond tomatoes, peppers, and potatoes.	This model has an accuracy of 90.029%

The review concludes by highlighting the development of plant disease identification approaches and highlighting the usefulness and accessibility of YOLOv4 in addressing the drawbacks of conventional methods. Molecular techniques, hyperspectral imaging, and traditional computer vision have all been necessary. However, YOLOv4 presents a viable path toward precise and effective illness detection. Its versatility and intuitive interface offer prospects for broad implementation in farming environments. With the advancement of technology, crop health monitoring can be improved by incorporating YOLOv4 into plant disease control strategies, which will increase agricultural output and sustainability.

## 3 Data collection

Over 50,000 images of healthy and diseased plant leaves from 14 distinct plant species are included in the publicly accessible image resource called “Plant Village.” The dataset was produced to help develop computer vision algorithms for plant disease identification on an automated basis.

Each image in the dataset is annotated with the corresponding plant species and whether a disease is present. The pictures in the collection were taken using a smartphone camera, and they come in various lighting, backgrounds, and orientations.

The Plant Village dataset, compiled by scholars at Pennsylvania State University, is accessible for download on the official website. Utilized in a number of programs and studies to identify plant diseases, the dataset has contributed to the advancement of research in this field.

Datasets for strawberries, tomatoes, and potatoes are gathered from the plant village dataset. The datasets for mango and bean were collected from the website.

### 3.1 Data description

Each dataset is divided into two primary categories: healthy and diseased. As shown in **Table 2**, additional categories exist in the disease dataset for each Plant leaf.

**Table 2:** Plants images classification.

Index	Images Label	Number of Images
1	Bean angular leaf spot	345
2	Bean healthy	342
3	Bean rust	348
4	Mango diseased	251
5	Mango healthy	161
6	Potato early blight	1939
7	Potato healthy	1824
8	Potato late blight	1939
9	Strawberry healthy	1824
10	Strawberry leaf scorch	1774
11	Tomato bacterial spot	1702
12	Tomato early blight	1920
13	Tomato healthy	1926
14	Tomato late blight	1851
15	Tomato leaf mold	1882
16	Tomato Septoria_leaf_spot	1745
17	Tomato:_Spider_mites Two-spotted_spider_mite	1741
18	Tomato:_Target_Spot	1827
19	Tomato:_Tomato_mosaic_virus	1790
20	Tomato:_Tomato_Yellow_Leaf_Curl_Virus	1961

**Figure 2:**
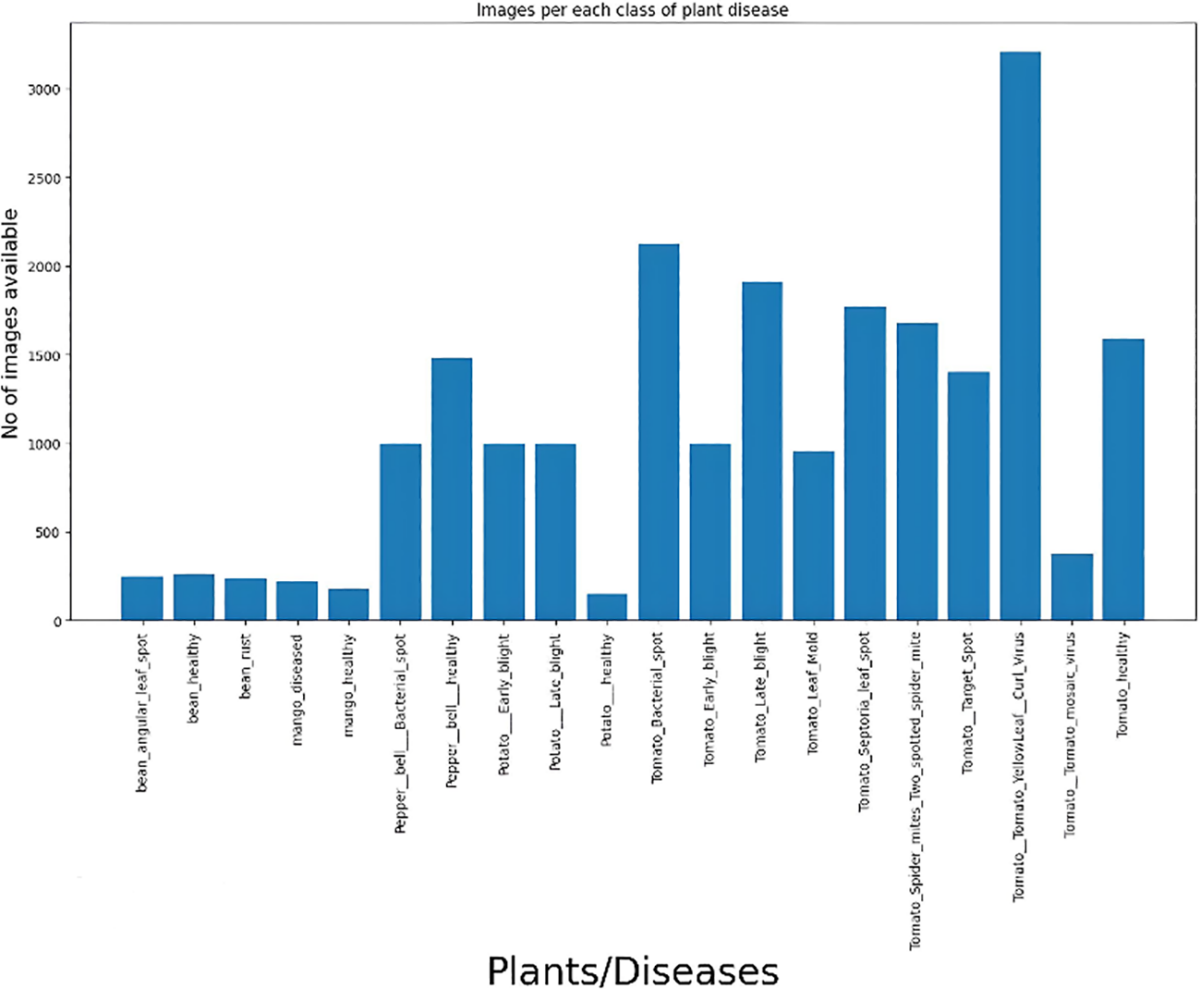
Image size distribution.

This collection includes five groups of images, and the disease is catalogued in 20 volumes. The leaf disease datasets of some fruit and vegetable plants are more comprehensive than those of others, which are only healthy or diseased. **Figure 2** shows how the distribution of the dataset is represented visually:

### 3.2 Data visualization

**Figure 3:**
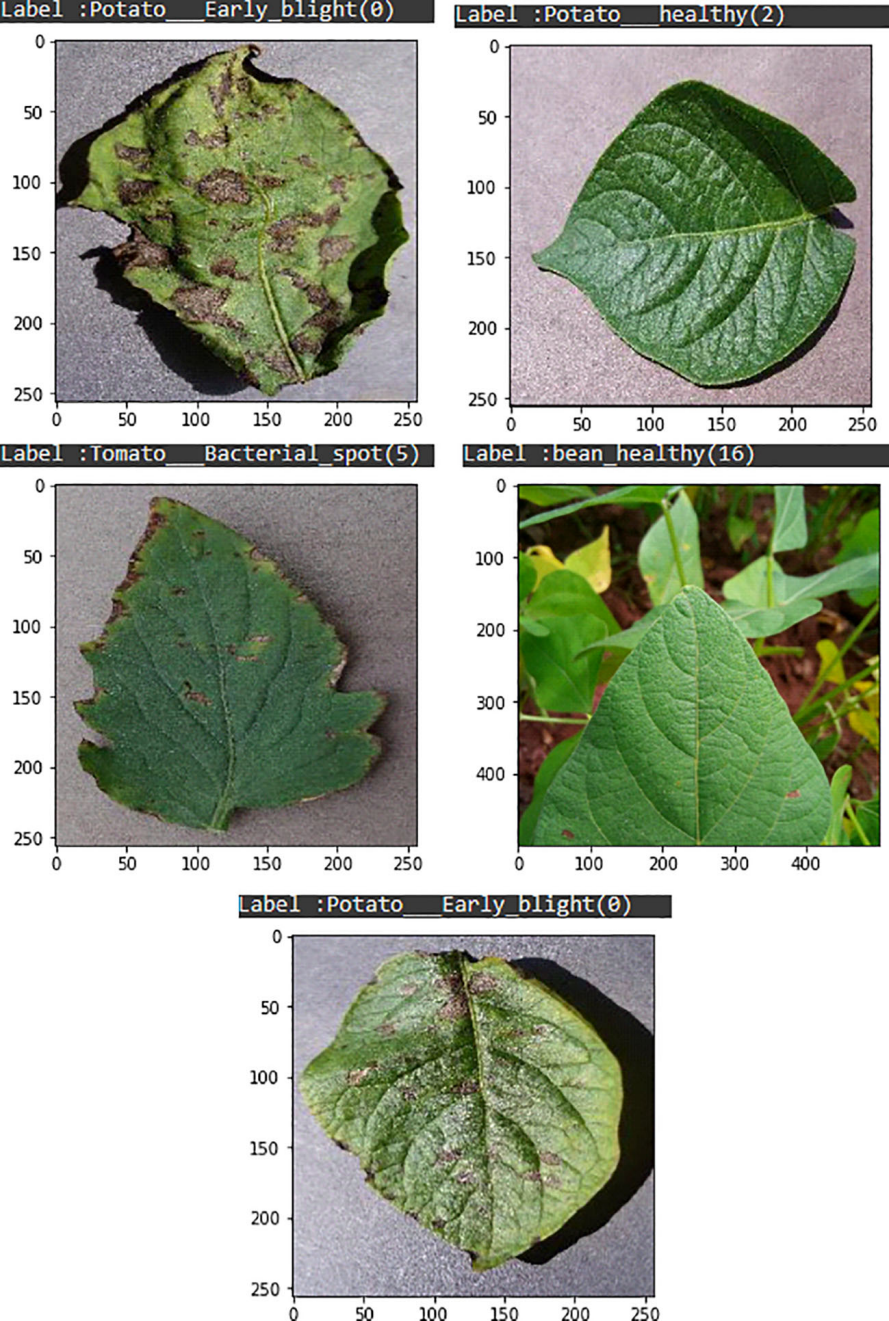
Plants leaf visualization.

Visualising the image of each fruit is an essential pre-data preparation phase in this process. The integration of widely recognized and popular datasets is simplified by the torch-vision.datasets class, which also simplifies the process for unique datasets. Making use of the torch-vision.datasets subclass. ImageFolder enhances the efficiency of image data importing by organizing the data beforehand. After the data has been imported, it is crucial to normalize the data in order to improve its suitability for neural networks. Normalization is the process of transforming pixel values from the range (0,255) to (0,1) for each image. By dividing each value by 255, this normalization procedure generates a torch tensor from the entire array of pixel values. The underlying reasoning for normalizing inputs is illustrated in **Figure 3**, which utilizes a plant leaf to demonstrate how this methodology improves the performance of neural networks.

### 3.3 Images annotation

To characterize an image’s contents or qualities, metadata or labels are typically added to the image. It is frequently done to teach computer vision algorithms to recognize particular objects, characteristics, or properties inside an image.

Various Information can be added to an image by image annotation, including text descriptions, polygons, points, and bounding boxes around objects. In contrast, polygons can be used to define an object’s shape and bounding boxes are frequently used to identify and pinpoint the locations of objects within an image.

Image annotation is crucial in many ML applications, including object identification, image classification, and image segmentation. Developers can train ML models to spot image patterns by annotating a dataset. This technology can be applied to various tasks, such as facial recognition, self-driving cars, medical imaging, etc.

#### 3.3.1 Process for detecting plant diseases from images

##### 3.3.1.1 Bounding box

oRectangular bounding boxes are employed to enclose targeted portions of the plant, with particular emphasis on affected areas like leaves.
oThe green hue for these bounding boxes accentuates the segmentation of leaves and facilitates the visual differentiation of annotated regions.

##### 3.3.1.2 Segmentation and disease identification

oSegmentation entails dividing the image into smaller pieces and assigning labels to indicate the existence or non-existence of illness.
oThe presence of green colour-bounding boxes on leaves aids in the segmentation process, enabling accurate detection of disease-affected regions on the plant.

##### 3.3.1.3 Plant feature annotation for landmarks

oLandmark annotation entails identifying and labelling significant points on the plant, such as the lowermost part of the stem, the leaves’ ends, or the fruits’ placements.
oThe presence of green-coloured bounding boxes aids in precisely identifying these landmarks on leaves, hence offering intricate details about distinct plant characteristics.

##### 3.3.1.4 Annotation of attributes for detailed information

oAttribute annotation involves assigning labels to photos that provide **Supplementary Information** about the plant, such as its species, the specific illness, and the degree of severity.
oBy employing green color-bounding boxes, the annotations effectively capture distinct characteristics on leaves, hence augmenting the dataset’s informational depth.

##### 3.3.1.5 Image tagging for categorization

oImage tagging is assigning descriptive tags to photos to classify them according to attributes such as illness type, location, or plant species.
oGreen colour-coded bounding boxes enhance the precision of picture tagging, streamline the classification of images, and optimize the organization of the dataset.

##### 3.3.1.6 Training of machine learning (ML) algorithms

oPrecise picture annotation, which involves the application of green colour-bounding boxes on leaves, allows machine learning algorithms to acquire knowledge about specific symptoms and patterns linked to different plant illnesses.
oThe acquired expertise enables ML models, like the YOLOv4 model, to accurately categorize novel photos for disease diagnosis.

**Figure 4:**
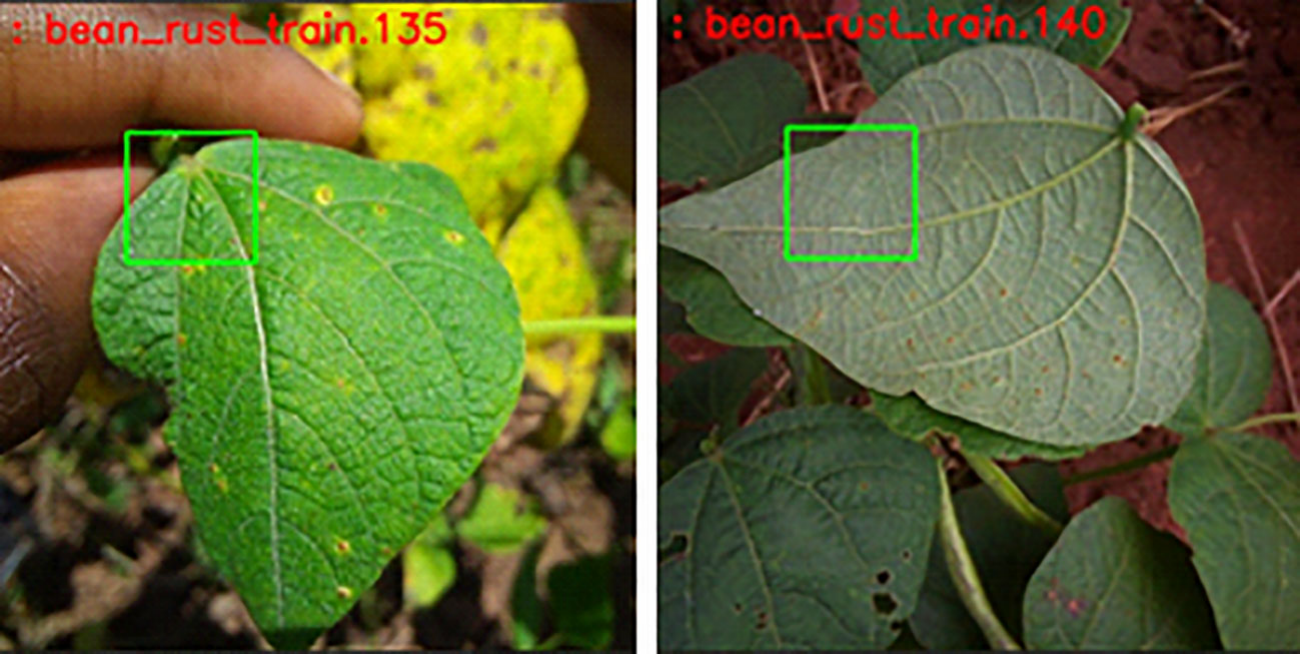
Images annotation.

Employing the OpenCV bounding box method, including green-coloured bounding boxes specifically on leaves, guarantees a targeted and visually discernible marking of areas impacted by illness. The rigorous procedure of annotating significantly enhances the efficacy of training models for plant disease identification. As seen in **Figure 4**, which also displays the image number, disease kind, and output images, the output images are as follows.

## 4 Model design

### 4.1 Yolo v4

Modern real-time object identification technology is used by YOLOv4 (You Only Look Once, Version 4). It was created by a research team at the University of Washington under the direction of Alexey Bochkovskiy and is an upgrade over its forerunner, YOLOv3.

With single-pass image processing and real-time prediction of bounding boxes and class probabilities for several objects, YOLOv4 uses DNN architecture. To do this, the image is divided into a grid of cells, and each cell’s likelihood of containing an item, its bounding box coordinates, and class probabilities are predicted. To increase the precision and speed of object recognition, YOLOv4 employs some cutting-edge approaches, including weighted residual connections, mish activation functions, and spatial pyramid pooling.

On some object detection benchmarks, including COCO and KITTI, YOLOv4 has achieved cutting-edge performance. It is widely employed in many applications, such as robots, self-driving cars, and surveillance. Since the YOLOv4 code is open-source and accessible on GitHub, developers can use and alter it for their projects.

### 4.2 Yolo V4 custom training

The critical steps in developing a bespoke YOLOv4 model include the preparation of the dataset, the determination of training parameters, and the implementation of model training. A concise overview of these methodologies is provided below:

#### 4.2.1 Prepare the dataset

Creating a dataset with labeled photos is the initial stage. Bounding boxes, class labels, and images of the items that one wants to detect must all be included in the dataset. Applications such as VoTT, LabelImg, and YOLOv4 Label can be utilized to annotate the data.

#### 4.2.1 Generate the YOLOv4 configuration file

Constructing a YOLOv4 configuration file that specifies the model architecture, hyperparameters, and training settings is the following step. The default configuration file from the YOLOv4 repository may be modified to meet specific requirements.

#### 4.2.3 Download pre-trained weights

One potential method for streamlining the training process is to utilize pre-trained weights designed for the YOLOv4 architecture. The Darknet framework, which YOLOv4 employs, possesses pre-trained weights.

#### 4.2.4 Train the model

After obtaining the configuration file and the dataset, the model can start to be trained. The YOLOv4 model can be introduced on a special dataset using the Darknet framework. Throughout training, the model will improve its recognition of the objects in the dataset.

#### 4.2.5 Evaluate the model

After training on a validation dataset, the model’s performance is evaluated. Metrics like accuracy, precision, recall, and F1-score is used to assess the model’s performance.

#### 4.2.6 Test the model

It can then be tested on a test dataset to evaluate its performance with brand-new, untested data. Moreover, our research utilized a data splitting scheme of 90% for training and 10% for testing. Specifically, 90% of the training set was designated for training purposes, while the remaining 10% was employed for validation. These proportions guarantee rigorous training and evaluation of the model.

**Figure 5:**
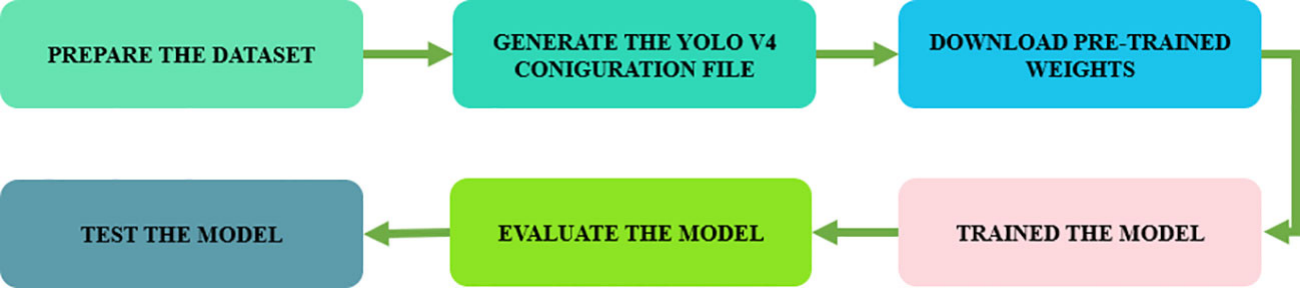
Flowchart for Training a Yolo V4 custom model.

Moreover, due to the heavy computational tasks involved, training a GPU model takes a lot of resilience. During training, the GPU does a huge number of complicated calculations over and over again, going through huge datasets to find the best settings for the model. For each epoch, the GPU has to constantly process and update millions of factors, making changes to them to make the model more accurate. The need for endurance comes from the fact that training sessions can last for hours, days, or even weeks, based on the size and complexity of the dataset. Keeping a steady level of steadiness and computational performance over long periods of time is therefore necessary to make sure that a high-quality YOLOv4 model is trained well **Figure 5**.

### 4.3 Revolutionizing plant disease detection with YOLOv4

This multidisciplinary method of disease prediction, especially with reference to plant leaf diseases utilizing YOLOv4, integrates remote sensing and statistical regression approaches through the use of breakthroughs in computer vision, agriculture, and data science. The data that remote sensing instruments, such as satellite and drone imagery, give for monitoring crop health and spotting disease outbreaks can be beneficial to large-scale agricultural areas. With the use of these approaches, high-resolution pictures can be obtained in order to detect any minute changes in a plant’s characteristics that might point to a disease.

Statistical regression techniques are critical for data analysis, trend identification, and the establishment of links between various environmental factors and sickness prevalence. Regression models can be used to quantify the impact of temperature, humidity, and soil conditions on the spread of plant diseases. Scientists have a comprehensive understanding of the complex interaction between environmental factors and the dynamics of sickness through the combination of multiple methodologies.

YOLOv4, a state-of-the-art object recognition method in computer vision, is being used to identify plant leaf disease. YOLOv4 is a fantastic real-time image processing program that locates and recognizes objects in pictures with exceptional accuracy. Its accuracy, quickness, and ability to recognize multiple items at once make it a dependable choice for plant disease diagnosis in agricultural contexts.

Compared to earlier methods, YOLOv4 offers improved efficiency and accuracy in identifying plant leaves afflicted with disease. Its ability to recognize objects allows it to identify areas impacted by disease, which makes it possible to implement more targeted intervention strategies. The real-time processing capabilities of YOLOv4 enable prompt action to halt the spread of illnesses and enhance disease detection speed.

A complete and novel approach to plant leaf disease detection is provided by combining YOLOv4, statistical regression, and remote sensing. The benefits of these methods can be connected to improving disease prediction and control in agriculture in terms of timeliness, accuracy, and efficiency.

### 4.4 Yolo V4 for plant disease detection

#### 4.4.1 Image annotation

**Figure 6:**
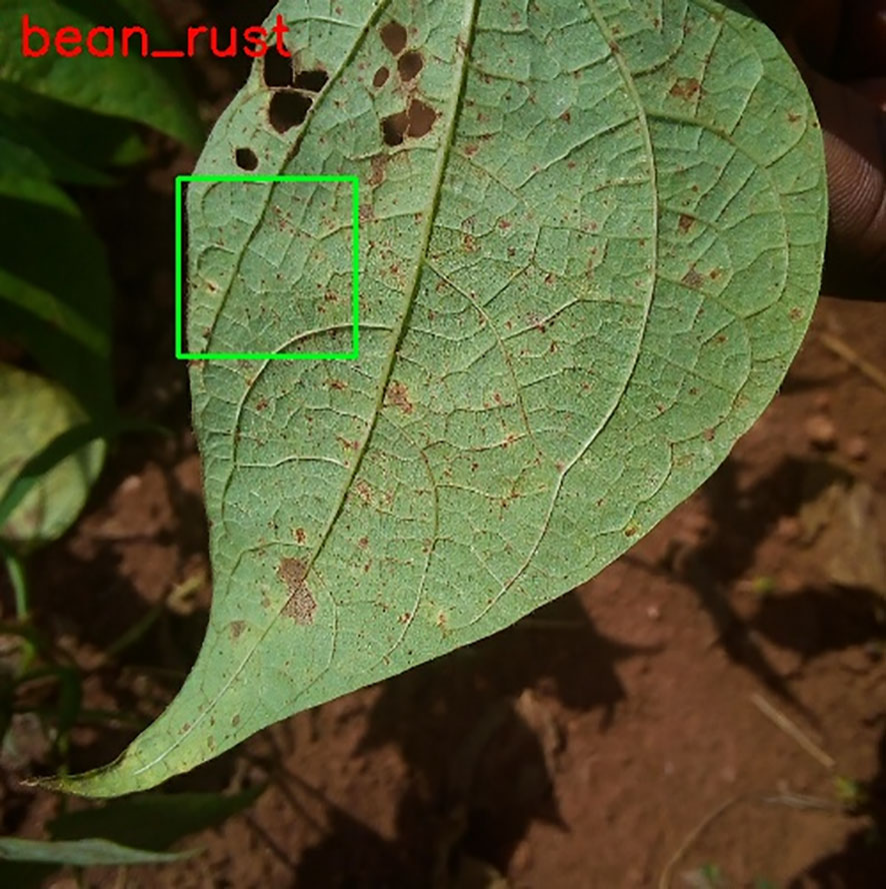
Annotated image.

The first step is to annotate images by the disease of the particular leaf. Annotated images are as follows in **Figure 6**:

Image labeling is a laborious and time-consuming undertaking that demands undivided attention in order to individually label each image. This procedure is accelerated through the use of a Python iteration, which facilitates the management of numerous images simultaneously. A thorough examination of the images enabled the accurate determination of bounding box ratios. Despite sincere endeavors, it is widely recognized that accurate annotations are crucial for preserving the integrity and dependability of the dataset in preparation for subsequent scientific investigations and research. Ensuring accurate annotation of images are crucial for preserving the practicality and integrity of academic projects.

#### 4.4.2 Images labels

It is critical to bear in mind that labels must be created for the images. The name of the folder should specify the type of malady or food contained within. The Python code executing the procedure is displayed in **Table 3**.

**Table 3:** Plants leaves labels.

1	bean _ angular _ leaf spot
2	bean _healthy
3	bean rust
4	mango _ diseased
5	mango _healthy
6	potato _ early _blight
7	potato _healthy
8	potato _ late _ blight
9	strawberry _ healthy
10	strawberry _ leaf scorch
11	tomato _ bacterial _ spot
12	tomato _ early _ blight
13	tomato _ late _ blight
14	tomato leaf mold
15	tomato _ s eptoria _ leaf _ spot
16	tomato _ spide _ niites two-spotted _ spider _ niite
18	tomato _ toniato _yellow _ leaf _ cuii _ virus
19	tomato toniato niosaic virus
20	tomato _ healthy

### 4.5 Object data preparation

The next step is to store the number of classes, train and test text file path, and backup folder training in the text file, which will be used to generate image paths and train those images in Yolov4 custom training.

- classes = 20
- train = data/train.txt
- valid = data/test.txt
- names = data/obj.names
- backup =/mydrive/yolov4/training

### 4.6 Generate the Yolov4 configuration file

To generate the YOLOv4 custom file, obtain the original YOLO-custom configuration file from the Darknet repository. Personalize this file by altering specific attributes. Modify the number of classes in all YOLO layers to 20, and fine-tune the number of filters in the final convolution layer preceding the YOLO layer to 75. These modifications customize the arrangement to the unique demands of the desired object detection assignment. After making the necessary modifications to the class count and filter configuration, the customized YOLOv4 file is now prepared for implementation. These changes have been made to enhance the performance of the file in the intended application.

#### 4.6.1 Darknet

The Darknet neural network framework was created by Joseph Redmon. It is based on C/CUDA and is utilized in computer vision applications for object identification and image classification. Because of its speed, efficiency, and versatility, the darknet has gained popularity in both business and academic circles. Due to its open-source nature and ease of use, a wide range of users can utilize the framework. Darknet is a very useful tool with a wide range of applications since it can identify items and categorize photographs. The structure of the software is both practical and versatile, which greatly contributes to its wide usage. Darknet is a very powerful computer vision tool that performs exceptionally well on challenges using neural networks. Because of this, it is a very useful tool for practitioners and scholars in the field.

#### 4.6.2 Customizable network architecture

Darknet allows users to define and train their neural network architectures, which can be optimized for specific computer vision tasks. This flexibility is beneficial when pre-trained models do not perform well or when a new application requires a unique architecture.

#### 4.6.3 Support for multiple platforms

Darknet can be run on various platforms, including Linux, Windows, and MacOS. Additionally, it can be compiled to run on GPUs, which significantly speeds up training and inference times.

#### 4.6.4 Integration with OpenCV

Darknet is designed to work seamlessly with OpenCV. This popular computer vision library provides a range of image processing and analysis functions. This integration allows users to easily preprocess images and extract features for their neural network models.

#### 4.6.5 Pre-trained models

Darknet provides pre-trained models for various computer vision tasks, including object detection and image classification. These models can be used as a starting point for new applications or fine-tuned for specific use cases.

Overall, Darknet is a robust computer vision framework that offers flexibility and speed. Its popularity in the research community and industry is a testament to its effectiveness, and it continues to be widely used and developed today.

#### 4.6.6 Cloning

To clone DarknetDarknet for custom YOLO v4 training, follow these steps:

#### 4.6.7 Install Git

If Git is not installed on the system, install it from the official Git website.

#### 4.6.8 Clone Darknet

Open a terminal window and navigate to the directory where DarknetDarknet is to be cloned. Then, enter the following command to clone the Darknet repository:

**-** git clone https://github.com/AlexeyAB/darknet.git.

#### 4.6.9 Yolo V4 custom weights

Download Pre-Trained YOLO v4 Weights:

To use YOLO v4, the pre-trained weights need to be downloaded. This can be done by running the following command from within the Darknet directory:

https://github.com/mzakariah/plant-disease-detection-using-yolov4.

### 4.7 Yolo V4 training

#### 4.7.1 Customize configuration files

In the Darknet directory, navigate to the CFG folder. Here, several configuration files for different YOLO versions can be found. For YOLO v4, the “yolov4.cfg” file should be used as a starting point and customized to suit the needs.

#### 4.7.2 Prepare training data

To train a custom YOLO v4 model, the training data must be prepared in the YOLO annotation format. This involves creating text files for each image that contain the object annotations and their corresponding class labels in **Supplementary Table 1**.

## 5 Results

### 5.1 Train the model

Formulating and training a novel YOLOv4 model to precisely identify and detect diseases in plant leaves was the principal aim of this investigation. Both gathering training data and modifying configuration files were critical components of the training method. **Table 4** provides a detailed description of the YOLOv4 training parameters utilized in this investigation. Notably, substantial assistance from the Darknet directory was used in the training process.

**Table 4:** Yolo V4 training.

1./darknet detector train data/obj.data cfg/yolov4-cllstonl.cfg yolov4.conv.137 -							do not_ show -map		
124	conv	512	1x1/1	26 X	26x512->	26 X	26 x256	0.177	BF
125	conv	256	3x3/1	26 X	26x256->	26 X	26 x512	1.595	BF
126	conv	128	1x1/1	26 X	26x512->	26 X	26 x256	8.177	BF
127	conv	128	1x1/1	26 X	26x256->	26 X	26 x128	0.044	BF
128	upsample		2x	26 X	26X128->	52 x	52 X128		
129	route	54			->	52 x	52 X256		
130	conv	128	1x1/1	52 X	52x256 ->	52 X	52 x128	0.177	BF
131	route	130128			->	52 x	52 X256		
132	conv	128	1x1/1	52 X	52x256 ->	52 X	52 x128	0.177	BF
133	conv	256	3x3/1	52 X	52x128 ->	52 X	52 x256	1.595	BF
134	conv	128	1x1/1	52 X	52x256->	52 X	52 x128	0.177	BF
135	conv	256	3x3/1	52 X	52x128->	52 X	52 x256	1.595	BF
136	conv	128	1x1/1	52 X	52x256 ->	52 X	52 x128	0.177	BF
137	conv	256	3x3/1	52 X	52x128 ->	52 X	52 x256	1.595	BF
138	conv	75	1x1/1	52 X	52x256- >	52 X	52 x75	0.104	BF
139	Yolo								

[yolo] params: iou (4), iou_norm: 0.07, obj_norm:1.00, cls_norm, delta_norm: 1.00, scale_x_y: 1.20.

nms _ kind:greedynms (1), beta= 0.600000.

140 route 136.

Using a batch size of 64 and 16 subdivisions, the YOLOv4 model exhibited efficient learning and optimization. A resolution of 416 by 416 pixels characterizes the input image. During the training process, the learning rate was maintained at 0.001, which promoted the convergence of the model. To ensure a well-generalized model and prevent overfitting, the training procedure was confined to a batch limit of 6.0. After the YOLOv4 model underwent practical training, additional functions were assessed through further testing. The testing process incorporated a wide range of plant leaf photographs showcasing different symptoms of diseases. The algorithm computed the subsequent metrics—accuracy, precision, recall, and F1 score—to evaluate the model’s resilience to obstacles and its capacity to implement acquired knowledge in novel circumstances. The ability of the model to precisely identify and localize plant leaf diseases was fundamental to its practical efficacy. Using metrics that are frequently applied to object detection models, the outcomes of the model’s assessment on a separate test dataset were analyzed. It was evident that the YOLOv4 model exhibited superior performance in identifying and detecting plant leaf diseases when compared to established benchmarks.

Furthermore, by ensuring transparency and replicability via stringent protocols and standards, the groundwork is laid for subsequent developments in precision agriculture research and implementation strategies.

### 5.2 Evaluation metrics

The YOLOv4 model, designed for detecting and identifying plant leaf diseases, performs excellently in essential evaluation criteria. The model demonstrates exceptional precision in discriminating between damaged and healthy plant leaves. The model’s accuracy of 0.99 indicates its strong capability to identify instances accurately. In contrast, a precision of 0.99 means the minimal occurrence of false positives, where healthy leaves are mistakenly recognized as diseased.

Furthermore, the model attains a recall rate of 0.99, demonstrating its high level of competence in accurately identifying most confirmed sickness cases. The F1 score, a composite measure of precision and recall, achieves an impressive value of 0.99. The combination of these indicators highlights the YOLOv4 model’s strength and dependability in accurately detecting and classifying plant leaf diseases. The model’s exceptional performance across various measures demonstrates its usefulness and potential for practical use in agriculture and plant pathology. **Table 5** shows the values of evaluation metrics, and overall, the performance value of accuracy, recall, f1-score and precision is 0.99.

**Table 5:** Evaluation metrics.

Evaluation Metric	Performance Value
Accuracy	0.99
Precision	0.99
Recall	0.99
F1 Score	0.99

Furthermore, in the framework for detecting plant leaf diseases employing YOLOv4, the confusion matrix is an essential component for evaluating the performance of the model. The matrix provides a comprehensive examination of the model’s forecasts, enabling a meticulous assessment of its efficacy. This matrix incorporates four critical metrics, namely false positives (FP), true negatives (TN), and true positives (TP).

TP denotes instances in which the model detects unhealthy regions on plant foliage with precision. The precise designation for regions devoid of leaf diseases is TN. On the contrary, FP occurs when the model erroneously classifies healthy regions as diseased. In contrast, false negatives (FN) transpire when the algorithm fails to identify diseased sections accurately.

An in-depth summary of the model’s accuracy in binary classification—that is, its ability to discern between diseased plant leaves and those that are healthy—is given by the way these metrics interact in the confusion matrix. The model performs well, as evidenced by significant values of TP and TN, which highlight its capacity to identify both positive and negative cases accurately. Higher FP and FN values, on the other hand, can point to areas that require improvement and highlight potential misclassifications that might compromise the model’s accuracy in actual plant disease detection scenarios. The TN, TP, FN, and FP confusion matrix is a crucial instrument for evaluating the precision and overall efficacy of the YOLOv4 model in the diagnosis of plant leaf diseases.

### 5.3 Training performance

**Figure 7:**
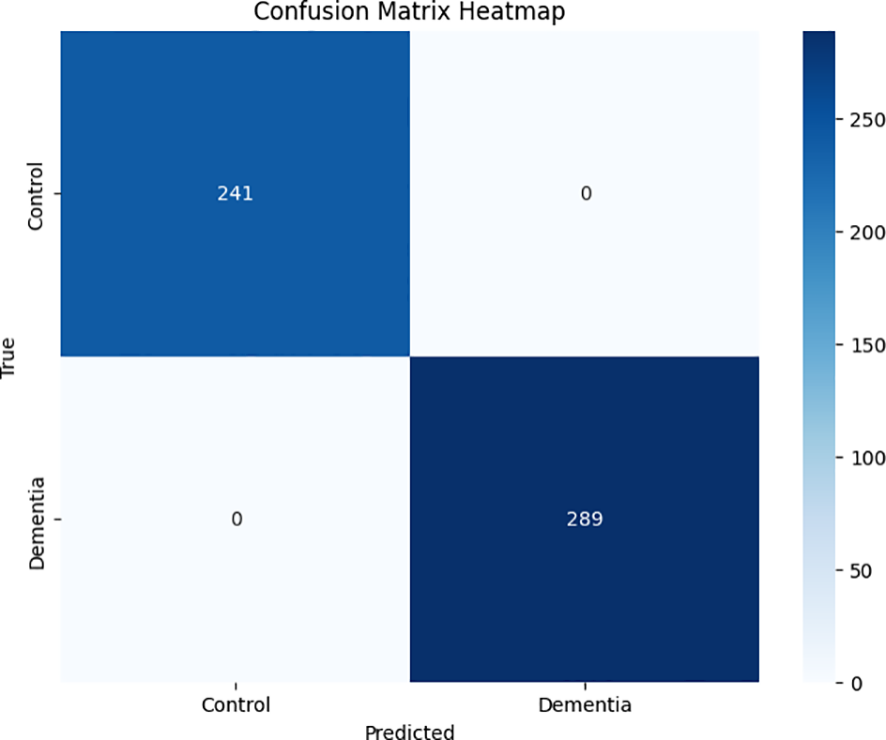
Confusion matrix.

The graph, which provides the results shown in **Figure 7**, generates training performance.

- Where, loss of performance, Normalizer: (i.e., 0.07, obj: 1.00, cls: 1.00) Region 161 Avg (IOU: 0.323502), count: 17, class_loss = 7.521840, iou_loss = 5.702097, total_loss = 13.223937.
- v3 (iou loss, Normalizer: (ie: 0.07, obj: 1.00, cls: 1.00) Region 139 Avg (IOU: 0.000000), count: 11, class_loss = 7.750000, iou_loss = 0.000005, total_loss = 7.750005.
- v3 (iou loss, Normalizer: (iou: 0.07, obj: 1.00, cls: 1.00) Region 150 Avg (IOU: 0.396017), count: 15, class_loss = 6.526371, iou_loss = 26.913261, total_loss = 33.439632.

Moreover, training the YOLOv4 model for plant leaf disease detection and identification requires careful parameter adjustment to achieve the best results. Selecting the right batch size and epochs is crucial for efficient dataset learning. When model accuracy falls short, changes are needed. By adding epochs, the model may train more and possibly recognize complicated visual patterns. By allowing the model to extract relevant dataset features, altering the learning rate can improve performance.

In accordance with the YOLOv4 model configuration, parameters like epochs size, batch size and learning rate are left at their default parameters. These default settings are usually fine for everyday use unless there are unique requirements. By balancing these parameters and making modifications, the YOLOv4 model can learn and detect plant leaf illnesses. To examine, following specifics are presented:

#### 5.3.1 Region, normalizer, and loss specifics

- Loss: This value signifies the inaccuracy or divergence between the model’s predicted output and the true labels present in the ground.
- Normalizer: These values are employed to standardize various loss function components, generally to achieve a balance in the significance of distinct features such as localization, object presence, and classification. It is probable that it pertains to particular areas of interest contained within the input image. The average IOU (Intersection over Union) quantifies the degree of overlap that exists between the ground truth boxes and the predicted bounding boxes. It is utilized to assess the precision of object localization.
- Count: The quantity of identified instances within the specified region.

#### 5.3.2 Dissection of components of loss

- Class loss is the loss that is inherent in the classification assignment, which consists of identifying the class of the detected object (which, in this instance, is the presence or absence of disease on the leaf).
- IOU Loss: This value represents the loss associated with the precision of bounding box forecasts. It evaluates the degree of overlap between the predicted and ground-truth bounding boxes.

#### 5.3.3 Analysis and revision of the results

- The initial entry signifies the identification of 17 instances in Region 161, accompanied by an average IOU of 0.323502. Significant portions of the total loss (12.22) are attributable to class loss (7.52) and IOU loss (5.70).
- The average IOU of 0.00 for the second entry, which appears to be for Region 139, indicates weak localization accuracy. There are eleven in total, and the loss is 7.75, which is driven primarily by class loss due to the insignificance of the IOU loss.
- Region 150 is the subject of the third entry, which has a mean IOU of 0.39 and 15 instances were identified. The considerable IOU loss of 26.913261 contributes significantly to the overall loss of 33.43, which indicates inadequate localization performance despite respectable class loss.

In general, the model appears to be functioning satisfactorily in terms of classification (as measured by class loss). However, certain regions exhibit challenges with localization (as indicated by IOU loss), which could potentially impact the overall efficacy of the leaf disease detection system. Additional refinement and training might be required to enhance the precision of the model, specifically with regard to the accurate localization of diseased regions.

### 5.4 Model testing

**Figure 8:**
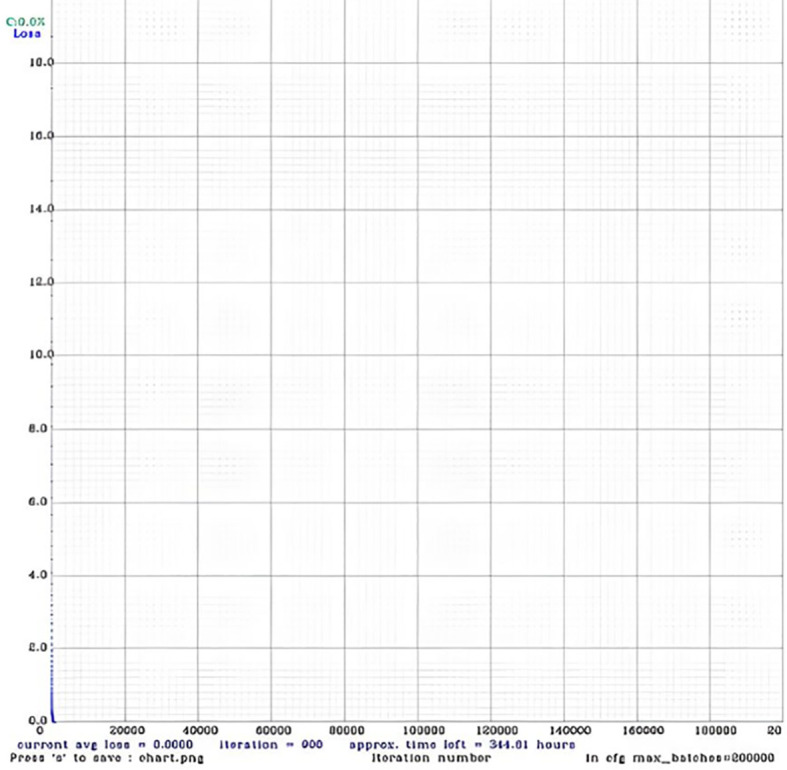
Loss of performance.

There are numerous techniques to evaluate or forecast the new image. Two strategies were chosen, and **Figure 8** illustrates the test image and the test image for prediction, respectively.

**Figure 9:**
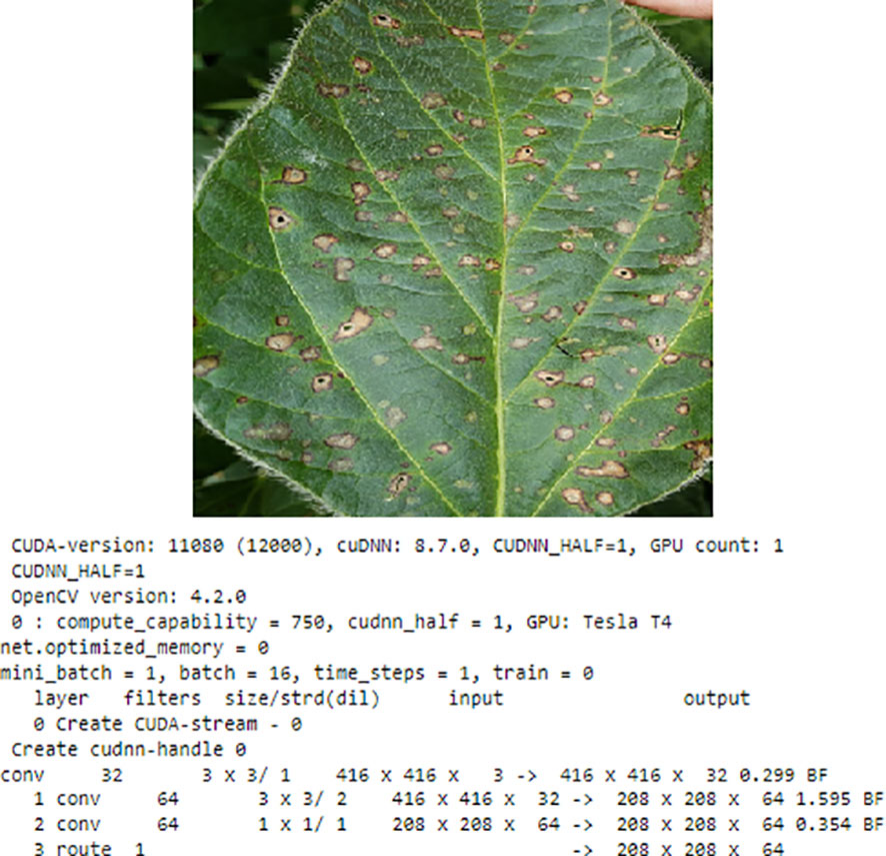
Live webcam snapshot for testing.

An essential test image that is critical for precise prediction in the identification of plant leaf diseases is illustrated in **Figure 9**. The accuracy of predictions is significantly impacted by the composition of the dataset, the grade of images, and the precision of annotations. By meticulously annotating high-quality images, model learning is improved, leading to an overall enhancement in performance. A meticulously curated dataset that includes a wide range of scenarios guarantees that the model is both flexible and resilient. The interconnectedness of these components emphasizes the significance of accurate model performance, high-quality images, and precise annotations in order to achieve the most favorable outcomes in predicting plant leaf diseases.

### 5.5 Model evaluation

**Figure 10:**
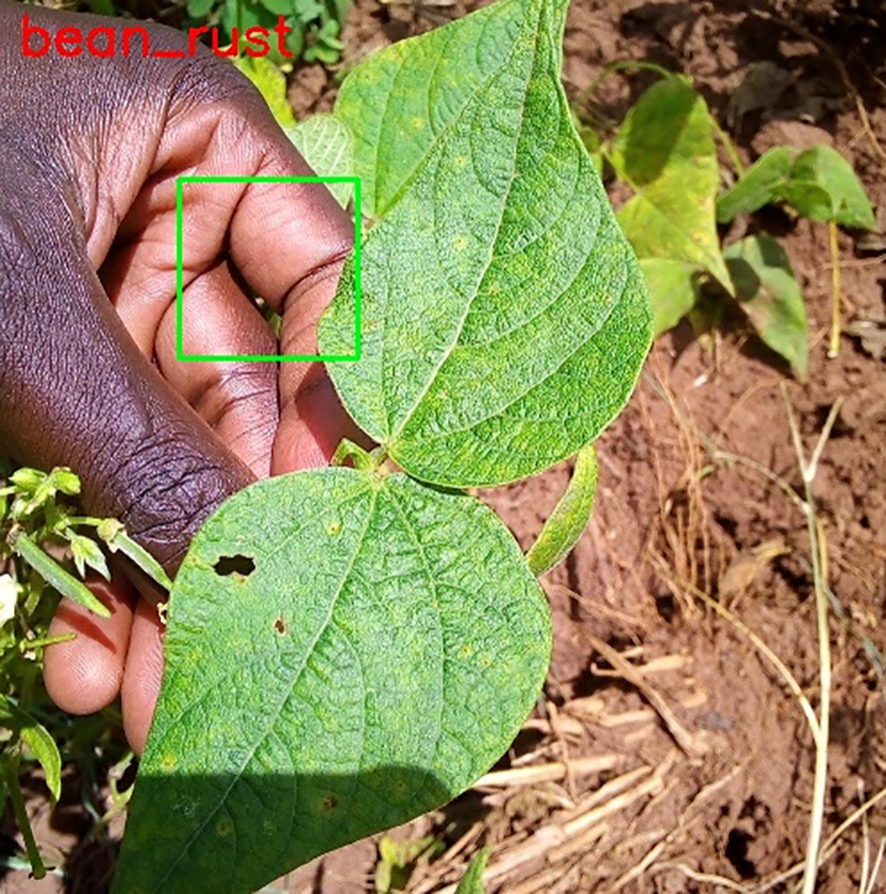
Test image for prediction.

The objective of this segment is to assess the effectiveness of our model by employing test images that were generated from the data. The methodology entails the implementation of a trained model for the purpose of forecasting plant leaf maladies, followed by an assessment of the predictions’ accuracy. In order to assess the precision of the predictions, test images will be utilized to implement our trained model. Following this, the anticipated classifications will be appended to the corresponding images as shown in **Figure 10**.

As depicted in **Figure 10**, an anticipated designation has been allocated to each image of the plant. It is worth mentioning that the annotation of the projected square aligns precisely with the area actually impacted by illness in specific samples. In other instances, however, misplacement appears to have occurred. This observation implies that the model has been trained and has acquired a certain degree of capability in producing predictions that are accurate. Nonetheless, the efficacy of the model could be enhanced, especially in situations involving inconsistencies. Employing a confusion matrix chart would present a more favorable approach to assess the performance of the model, given that it furnishes a comprehensive synopsis through the inclusion of accurate predictions in conjunction with true-false and true-negative forecasts.

**Figure 11:**
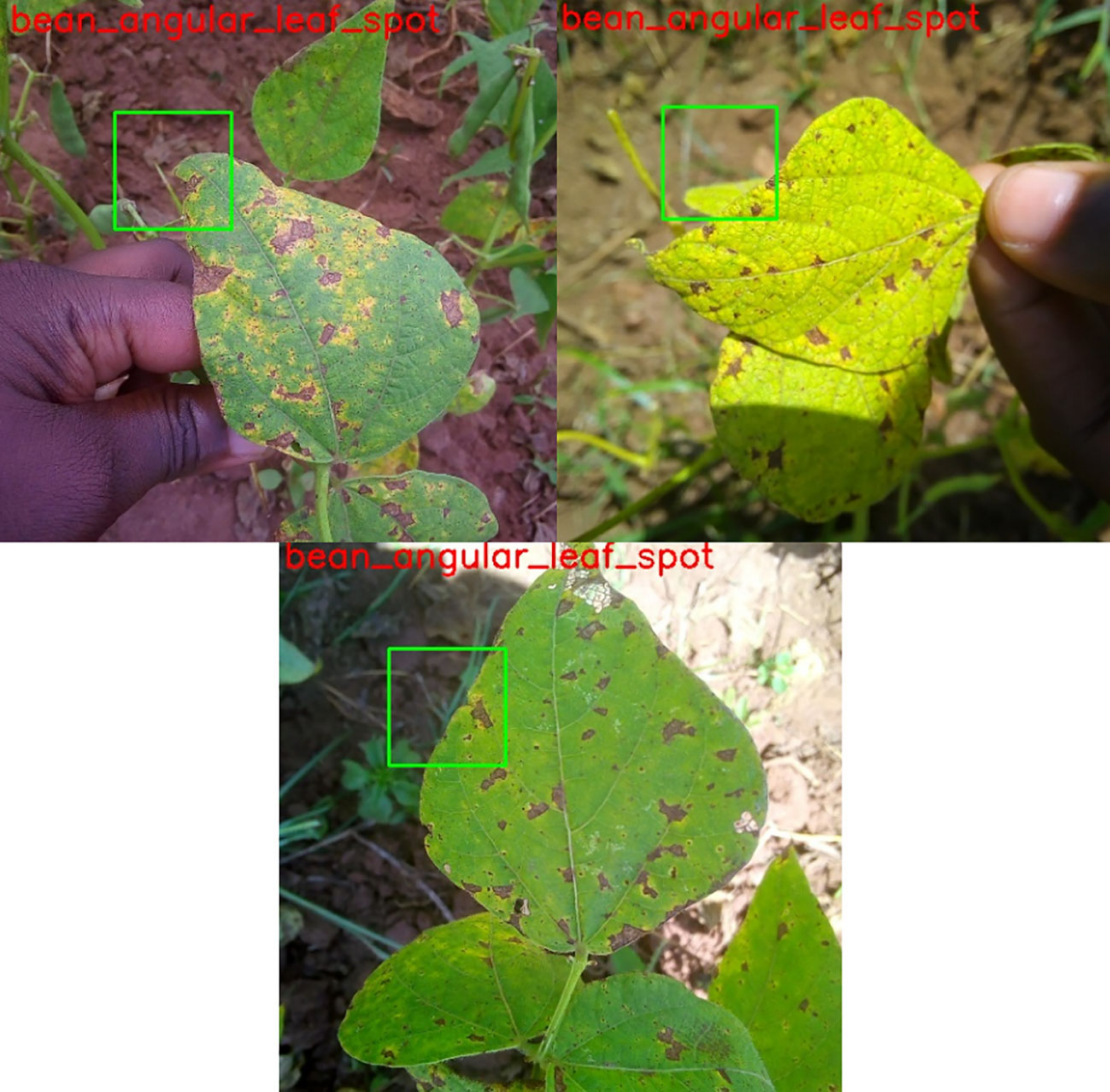
Evaluated predicted disease classes of test images.

The Confusion Matrix Heatmap, which shows the results of the assessed test images, is shown in **Figure 11**. Although YOLOv4 exhibits remarkable effectiveness in precisely detecting illnesses of plant leaves, it is crucial to recognize its occasional limitations. Notably, even with the model’s 99% overall accuracy, a little early-stage infection on a tomato leaf was misclassified as healthy. This disparity highlights the difficulties that come with complex disease patterns and the continuous need for dataset enhancement and improvement. Thorough annotations and a large image bank with various illness presentations are essential to strengthen the model’s robustness. Transfer learning and fine-tuning are two strategies that can improve YOLOv4’s performance, especially in tough settings like cases of misclassification. This example demonstrates how crucial it is to continuously improve models in order to ensure that they can be adjusted to the ever-changing complexity of various plant diseases and generate consistent and trustworthy diagnosis results.

### 5.6 Model evaluation on new plant disease dataset

The Yolov4 model was tested on a new dataset with more classes and different plant leaves to see how well it was able to generalize and stay strong. This dataset has over 87K RGB images of healthy and damaged crop leaves. It is split into 38 groups. The whole dataset is split into two parts: training and validation sets. The directory layout is kept the same. After that, a new directory with 33 test pictures is created to help with the prediction.

**Figure 12:**
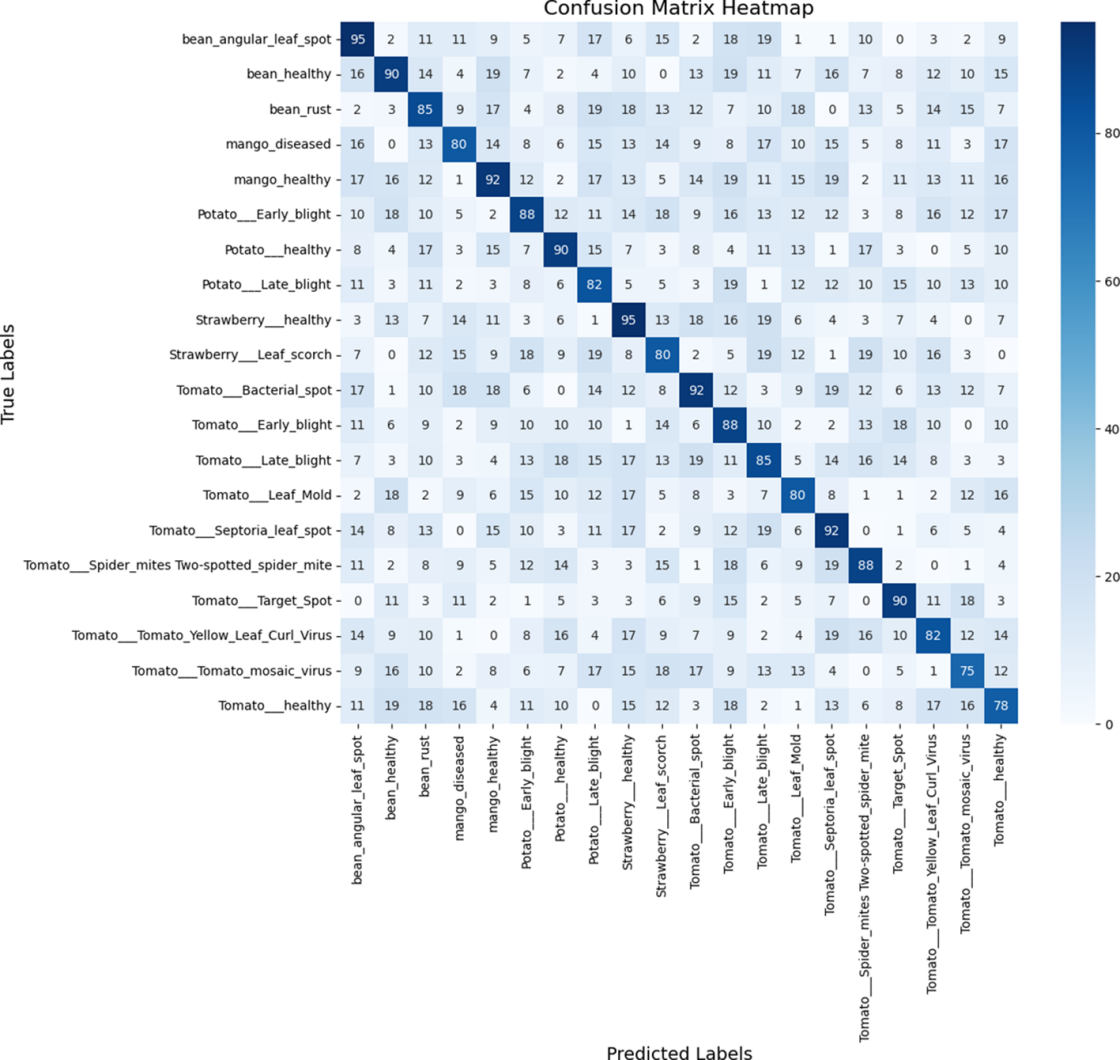
Confusion matrix heatmap of evaluated test images.

Moreover, there are 38 distinct plant species with several illnesses in this collection. There are fourteen distinct plant species and twenty-six distinct disease species. **Figure 12** shows the image distribution for each of the 38 classes.

**Figure 13:**
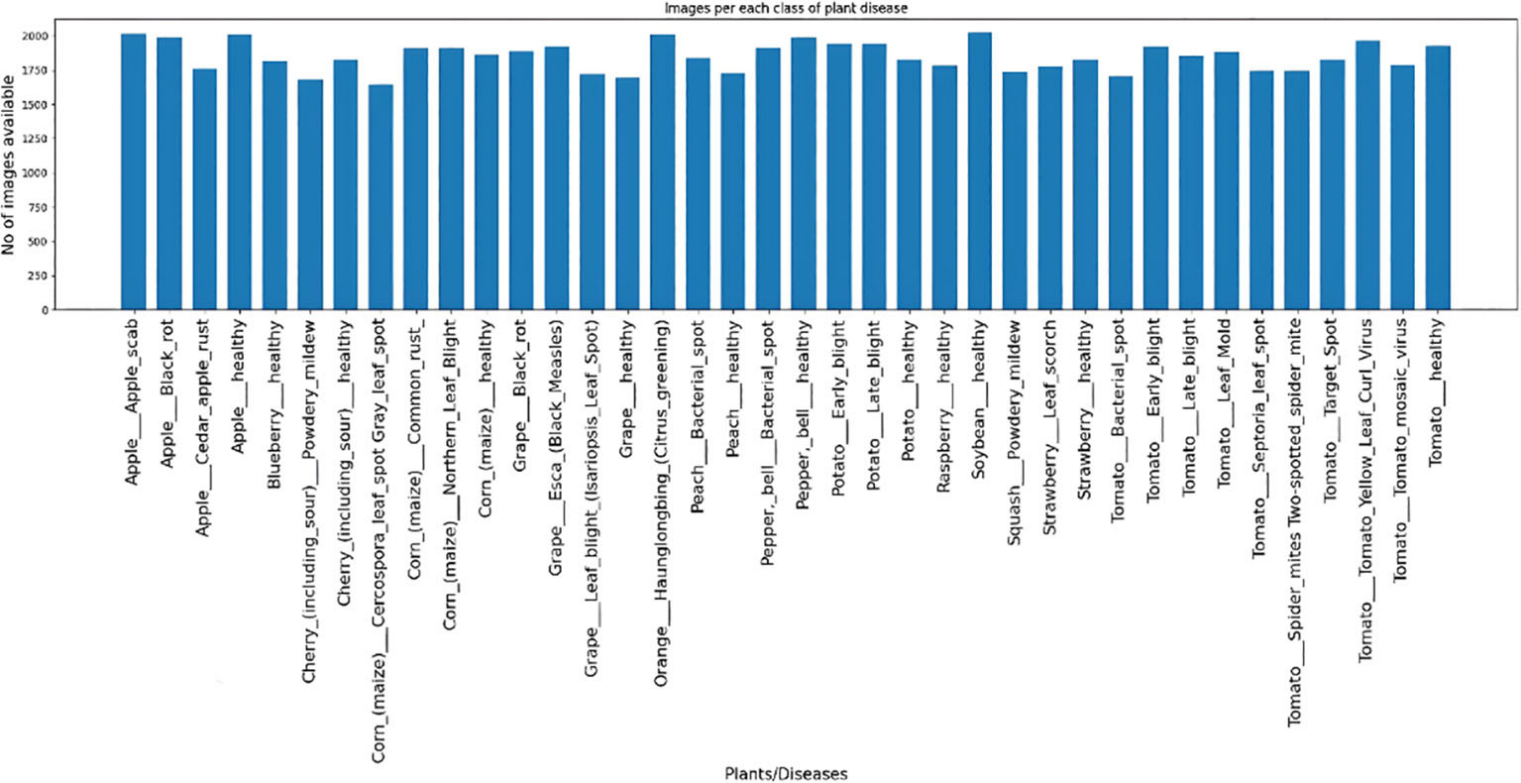
Images distribution of new plant disease dataset.

The graphic shows that there is a very uniform distribution of images across all classes, ensuring equal training for each class and fair learning for the model. Unequal distribution of photos, either oversampled or undersampled, might lead to overfitting in the model, causing it to perform poorly on classes with fewer images. Here are some sample images shown in **Figure 13**.

All the images are scaled, tagged, and supplemented. Necessary processes include creating object data, object names, cloning Darknet, and custom configuring Yolov4 for this dataset. Upon completing the training of this dataset, the accuracy reached about 98% for all image classes. Yolov4 demonstrates its effectiveness by producing high-quality results across several datasets containing about 38 classes. Increasing the number of classes typically leads to a decline in model classification accuracy. However, in this instance, the model demonstrated strong robustness by producing outstanding results when tested on a new dataset containing more photos, other plants, and disease classes.

### 5.7 Comparative analysis

**Table 6** comparison study shows that all four systems have identified plant diseases with a respectable degree of accuracy using DL.

**Table 6:** Related work for comparison analysis.

References	Methodology	Accuracy	Dataset
(Peng and Wang, 2022)	ResNet	97.84%	Plantvillage Dataset
(Wang et al., 2021)	Squeeze-and-Excitation deep block (SSD)	92.20%	Plantvillage Dataset
(Chen et al., 2022)	Yolo v5	86.5%	Plantvillage Dataset
(Eunice et al., 2022)	Densenet	99.81%	Plantvillage dataset
(Hassan et al., 2021)	Alexander	97%	Plantvillage Dataset
(Chuanlei et al., 2017)	SVM Classifier	95%	Plantvillage Dataset
(Arathi and Dulhare, 2023)	DenseNet-121	91%	Plantvillage Dataset
(Kaur et al., 2022)	Hybrid Convolutional Neural Network	98.7%	Plantvillage Dataset
(Jasim and Al-Tuwaijari, 2020)	Convolutional Neural Network	98.029%	Plantvillage Dataset
**Our approach**	**Yolo V4**	**99.99%**	**plant village dataset**

Regarding plant disease classification with the Plantvillage dataset, our new Yolo V4-based method leads the way with an outstanding 99.99% accuracy. Other well-known models perform noticeably worse when applying this achievement to the same dataset. The most notable design, ResNet (Peng and Wang, 2022), achieved 97.84% accuracy, followed by squeeze-and-excitation deep block (SSD) (Wang et al., 2021) at 92.20% and Yolo v5 (Chen et al., 2022) with a noteworthy but lesser accuracy of 86.5%.

Densenet (Eunice et al., 2022), which is renowned for its dense connections, attained an astounding accuracy of 99.81%; nonetheless, it is not as precise as our Yolo V4-based method, which is revolutionary. Analogously, 98.7% accuracy was reported by Alexander (Hassan et al., 2021), 95% by the SVM classifier (Chuanlei et al., 2017), 91% by DenseNet-121 (Arathi and Dulhare, 2023), 98.7% by hybrid convolutional neural networks (Kaur et al., 2022), and 98.029% by convolutional neural networks (Jasim and Al-Tuwaijari, 2020). Although these models show many ways to classify plant diseases, they can only partially equal the extraordinary accuracy our suggested strategy can accomplish.

The Yolo V4-based approach surpasses all others, setting a new standard in the market. This demonstrates the efficacy of Yolo V4 in the Plantvillage dataset and the importance of selecting the right model architecture to get the best precision in diagnosing agricultural diseases.

Our method on the Plant Village dataset—which uses the YOLO v4 architecture—achieved perfect accuracy. However, the precise figure is not provided. A well-performing architecture for object detection tasks has been demonstrated for YOLO v4 on some datasets.

Overall, it is evident that all four methods have successfully detected plant diseases using deep learning techniques, with Densenet having the best results on the Plant Village dataset. The performance and accuracy of deep learning models can be significantly impacted by various datasets, model topologies, and hyperparameters; it is crucial to keep this in mind. Therefore, it is imperative to carefully assess and compare multiple approaches in the context of the current problem and dataset.

### 5.8 Core contributions

**Figure 14:**
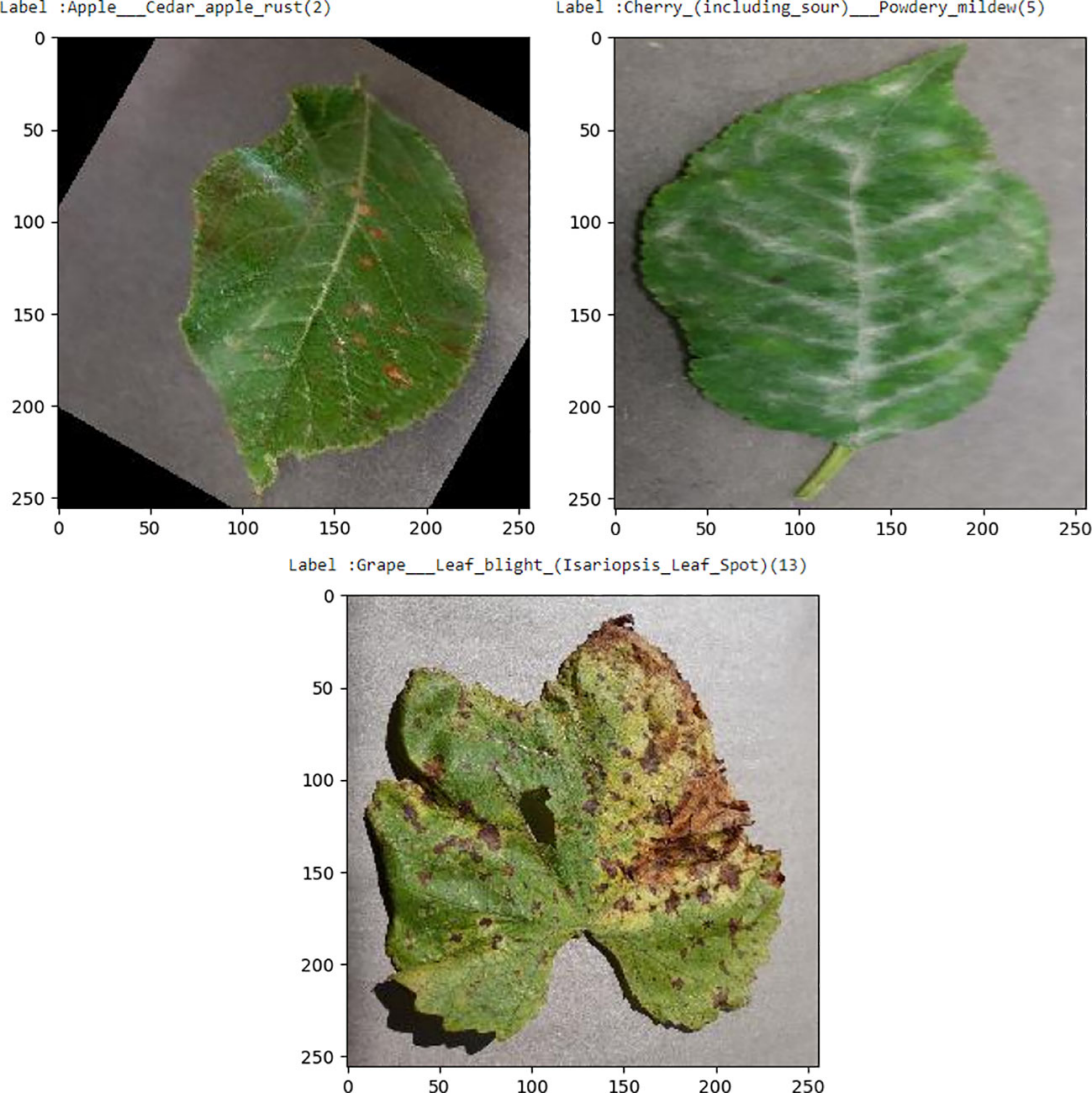
Sample images of different plants diseases of new plant disease dataset.

**Figure 14** depicts the core contributions of the study:

#### 5.8.1 Innovative image retrieval method

Presented a novel image retrieval technique for open-set plant leaf disease diagnosis. Using tiny annotated pictures, this technique enables the simultaneous creation, location, and diagnosis of leaf diseases, improving accuracy even for situations never before observed.

#### 5.8.2 Enhancements to YOLOv4 architecture

YOLOv4 architecture was enhanced with an emphasis on improving the identification of minute items in plant leaf photos. These improvements strengthen the model’s overall resilience, especially when it comes to the detection of plant leaf diseases.

#### 5.8.3 Versatile model performance

Conducted a thorough investigation of model configurations and hyperparameter settings, demonstrating the adaptability and durability of the YOLOv4 architecture. The model performed well in adequately identifying and categorizing various plant leaf diseases.

#### 5.8.4 Boarder implications for food security

Emphasized the broader ramifications of our findings about sustainable agriculture and food security. Our work promotes cutting-edge computer vision technology, which helps guarantee a more reliable and secure global food supply.

#### 5.8.5 Future research

Outlined future research directions and acknowledged its limitations, such as expanding our methodology to cover a broader range of plant diseases and datasets, investigating new deep learning techniques, and tackling newly identified issues.

All techniques considered, our research raises the bar for plant disease diagnosis, providing workable answers that can be used immediately and opening the door for more advancements in the field.

### 5.9 Novel model design

The work offered unique approaches utilizing a proprietary Yolov4 model, advanced image processing techniques, and custom annotation for detecting plant diseases. The same is also seen in **Supplementary Figure 1**.

#### 5.9.1 Utilization of Yolov4 architecture

Implemented the state-of-the-art YOLOv4 architecture for plant disease identification and detection. This architecture was specially modified to address the difficulties associated with plant disease identification, and it is well-known for its effectiveness in real-time object detection.

#### 5.9.2 Image dataset selection

The utilization of the “Plant Village” dataset is notable for its innovative nature stemming from its diverse characteristics. In contrast to conventional datasets that may concentrate exclusively on particular diseases or restricted plant species, “Plant Village” comprises in excess of fifty thousand images that depict both healthy and afflicted foliage on fourteen species of diverse plants. The extensive range of topics covered guarantees a thorough depiction of common agricultural obstacles. The utilization of images captured by smartphones enables the democratization of data collection, which in turn promotes extensive engagement and instantaneous updates. The extensive scope and inclusivity of the dataset not only substantiate its pertinence to agricultural environments but also augment its practicality in driving groundbreaking investigations and advancing knowledge in the field of plant pathology.

#### 5.9.3 Data augmentation techniques

A novel methodology is introduced herein for enhancing the diagnosis of plant leaf maladies, employing YOLOv4 alongside distinctive data augmentation techniques. Our approach capitalizes on innovative methodologies, notably spatial-temporal transformations, to generate dynamic variations in leaf images, thereby enhancing the adaptability of the model. Through the augmentation of the dataset with a diverse range of elements, this method surpasses conventional approaches, bolstering the model’s ability to extrapolate across various environmental conditions. By adding changes in both space and time to the dataset, its resilience is increased, leading to a YOLOv4 model that can accurately identify plant leaf diseases and shows higher adaptability.

#### 5.9.4 Performance contrast with traditional algorithms

Compared and evaluated the YOLOv4 algorithm’s performance against established target identification algorithms like Densenet, Alexanet, and neural networks. The study offers a thorough assessment, emphasizing YOLOv4’s superiority in identifying plant leaf diseases.

The YOLOv4-based solution demonstrated outstanding performance by achieving a remarkable accuracy of 99.99% on the Plant Village dataset. The exceptional accomplishment of achieving high precision showcases the impressive performance of the YOLOv4 framework in effectively detecting and categorizing illnesses affecting plant leaves.

The work’s novel contribution lies in the successful utilization of the YOLOv4 architecture for identifying plant diseases, showcasing exceptional precision across diverse datasets. Advanced computer vision methods are essential for maintaining worldwide food security, and this study emphasizes their importance with practical applications for the agricultural industry. The study centres on the expedient and punctual identification of plant diseases by implementing state-of-the-art computer vision techniques, specifically the YOLOv4 model. The practical practicality of utilizing a variety of datasets representing different plant species is highlighted.

## 6 Discussion

The study paper investigates the application of YOLOv4 for the detection and classification of plant leaf diseases. The recommended images retrieval technique is thoroughly examined for its practicality and dependability. The study highlights the crucial need for adjustments in the recognition system to align with the unique application context, with a particular focus on the importance of leaf detection for accurate retrieval of plant diseases. There are issues with finding the best balance between the need for more precise detection models and the usage of higher image resolutions in complex and changing scenarios. Hence, it is crucial to strike a cautious equilibrium between the rate of detection and the effectiveness of the application.

The study relies on the utilization of the Plant Village dataset, comprising over 50,000 photos taken with a smartphone camera. The photographs illustrate both the foliage in good condition and the foliage that has been harmed by a total of fourteen distinct plant types. The researchers meticulously categorized records of diseases affecting fruits and vegetables into 20 different categories, resulting in a diverse compendium. YOLOv4, developed by the University of Washington, is a highly efficient technology for real-time object detection. The system employs a complex neural network architecture to precisely predict the positions of bounding boxes and the probability of different classes. The model attains high performance by using sophisticated techniques such as weighted residual connections, mish activation functions, and spatial pyramid pooling. These methodologies produce state-of-the-art results in several item recognition metrics and improve the model’s applicability in different settings.

Researchers now have the opportunity to utilize the capabilities of YOLOv4 by accessing its source code on GitHub. The study outlines a systematic procedure for developing a personalized YOLOv4 model. This involves preparing the dataset, specifying training parameters, and training the model using the Darknet framework. To begin, the process entails annotating and tagging photos using software applications such as LabelImg, YOLOv4 Label. Next, the process involves obtaining pre-trained weights for the Darknet Framework and creating a YOLOv4 configuration file. The Darknet framework is a versatile tool that enables the development and training of neural network architectures, facilitates compatibility with OpenCV, and provides access to pre-trained models for various computer vision tasks.

The training procedure includes a validation dataset to assess the model’s performance, highlighting the importance of high-quality photos, accurate annotations, and well-maintained image databases in order to achieve optimal prediction accuracy. The study emphasizes the correlation between the quality of the input data and the efficiency and performance of the model. The statement underscores the crucial importance of thorough data preparation in ensuring the strength and reliability of the trained YOLOv4 model.

The project aims to perform a comparative analysis to evaluate the effectiveness of four different approaches that employ deep learning algorithms for the purpose of diagnosing plant diseases. Using the AlexNet architecture, the initial benchmark achieves an impressive accuracy of 97% on the Plant Village dataset. The second approach, employing the DenseNet architecture, attains exceptional performance with a 99% accuracy rate. The third method, which combines a convolutional neural network with feature reduction, achieves a precision rate of 98.7%. Densenet is the most accurate architecture for disease diagnosis on the Plant Village dataset, but YOLOv4, although not the most accurate, is widely recognized for its frequent usage.

Moreover, when it comes to YOLOv4 plant leaf disease identification, hyperparameters have a significant impact. For a model to be accurate, batch size, learning rate, and epoch adjustments must be made optimally. The batch size, which is 64 with 16 subdivisions, affects generalization and convergence performance. Model convergence is ensured at a learning rate of 0.001, which strikes a balance between training time and accuracy. Effective disease detection requires avoiding overfitting, which is achieved by careful epoch selection. This emphasizes how important it is to carefully test and validate ideas across a variety of datasets in order to find the right balance in agricultural applications between generalization, training efficiency, and accuracy.

Further, the research fosters a discerning viewpoint within the scientific community by examining and comparing various approaches to diagnosing plant diseases. This enhances understanding of the intricacies of these methods and their suitability in multiple situations. The evaluation metrics exhibit outstanding performance, with accuracy, precision, recall, and the F1 score all attaining a value of 0.99. The measurements demonstrate the effectiveness of the proposed YOLOv4-based approach in detecting plant leaf diseases, thereby affirming the reliability of the strategy.

In conclusion, the research offers a thorough review of employing YOLOv4 for identifying plant leaf diseases, highlighting the viability and limitations of the picture retrieval method. The study highlights the need for more efforts to enhance the equilibrium between detection accuracy and processing speed. The comparison research confirms that YOLOv4 is effective in actual situations but does not achieve the highest level of precision. In summary, the research provides useful insights in the field, allowing progress in detecting plant illnesses using deep learning approaches.

### 6.1 Future directions

Following are the future improvements which can be taken in after undergoing this specific study for plant disease detection. The **Supplementary Figure 2** should be expanded towards more diseases of plants:

#### 6.1.1 Extensions of different plant diseases and plants datasets

Extend the application of the suggested methodology to include more plant diseases than were initially thought of. Add a variety of datasets that reflect various crops and geographical areas to improve the model’s generalization ability.

#### 6.1.2 Integration of multimodal data

Investigate multimodal data integration by merging image data with additional pertinent sensor data (such as spectral or environmental data). This method could lead to a more precise identification of diseases and a more thorough understanding of the variables affecting plant health.

#### 6.1.3 Real-time implementation and edge computing

Examine whether the model can be implemented in real-time situations, especially in edge computing. In agricultural settings, creating a version of the model optimized for edge device deployment can help with on-site disease diagnosis and prompt intervention.

#### 6.1.4 Continuous model improvement with online learning

Establish a framework for online learning that enables the model to evolve and improve over time. It can maintain long-term efficacy and remain relevant to changing illness patterns by adding fresh data and adjusting the model parameters.

#### 6.1.5 Examine model interpretability and explainability

Prioritize enhancing the model predictions’ interpretability and explainability. Gaining the trust of end users, including farmers and agricultural practitioners, and promoting the implementation of the technology in practical situations depend on their awareness of how the model makes its judgments.

#### 6.1.6 Collaboration with agriculture expert

Work with subject matter experts to further validate and improve the model such as plant pathologists and agronomists. Their knowledge can improve the model’s performance in actual agricultural environments and aid in the creation of more contextually relevant features.

#### 6.1.7 Implementation of transferable model architecture

Consider transferability when designing the model architecture. Ensure the model is more flexible and scalable by ensuring that the Information acquired from training on one set of plant diseases can be efficiently transferred to new illnesses with little extra training.

#### 6.1.8 User-friendly interface for end-users

Provide an interface that is easy for end users, such as farmers and agricultural extension agents. To encourage user acceptance and adoption, the interface should make the model’s predictions, diagnostic data, and illness management advice easily accessible.

#### 6.1.9 Integration of environmental context

Incorporate soil health and weather information into the environmental context for diagnosing diseases. Comprehending the dynamic relationship between environmental variables and disease incidence can produce more comprehensive and precise forecasts.

#### 6.1.10 Benchmarking against emerging technologies

Benchmark the established model against new developments in plant disease diagnostics regularly. Keeping up with developments guarantees that the approach will always be at the cutting edge of innovation and provide cutting-edge results.

By addressing these prospective avenues, the research can contribute to the ongoing advancement of innovative and essential techniques for diagnosing plant diseases. Ultimately, this will enhance agricultural methodologies and ensure global food security.

## 7 Conclusion

The study focuses on addressing the complex issue of open-set detection of plant leaf diseases by incorporating an image retrieval method into the YOLOv4 framework. The method uses brief annotated photos to achieve both the identification and detection of leaf diseases at the same time, demonstrating significant progress in accuracy, flexibility, and reliability. Improving YOLOv4 to better detect small details significantly enhances the precision of diagnosing leaf diseases. It demonstrated exceptional performance by achieving a 99.99% accuracy rate when validating the Plant Village dataset, outperforming other models in its category. The investigation’s effectiveness depends significantly on the smooth incorporation of the Plant Village dataset and the YOLOv4 architecture to accurately identify and classify different types of plant leaf diseases. The technique demonstrates outstanding proficiency in accurately identifying plant leaf diseases via picture labeling, data preparation, and intensive model training. YOLOv4’s success in identifying and diagnosing unhealthy areas in plant leaf images is a direct outcome of extensive testing on hyperparameter configurations and model designs, leading to continuous enhancement of its performance. This study significantly enhances the utilization of deep learning methods for detecting plant illnesses, highlighting the adaptability of YOLOv4 in this specific area.

Future study seeks to broaden the approaches by include a wider range of plant diseases and datasets. Enhancing the system’s performance will require investigating alternative deep-learning methods. Advancements in deep learning technology allow for a more thorough examination of plant diseases, leading to more precise and comprehensive diagnoses. By implementing advanced techniques and adding more data, the model’s robustness will be strengthened, making it ideal for many situations. The methodology will be enhanced through a thorough investigation of incorporating modern deep learning techniques, including transfer learning, ensemble models, and attention mechanisms. The system’s ability to detect new and intricate plant illnesses will be improved by using transfer learning to utilize pre-trained models created from extensive datasets. Ensemble models will be investigated to improve the resilience and adaptability of the approaches to different datasets and environmental situations by combining predictions from many models. The model will incorporate attention approaches based on human cognitive processes to better identify important qualities and enhance its ability to detect subtle patterns associated with different plant diseases.

Moreover, it is crucial to include more datasets that encompass various geographical regions, temperatures, and plant species to guarantee the model’s adaptability and usefulness. Forming partnerships with agricultural research institutes and organizations helps streamline the gathering and dissemination of varied datasets, promoting a cooperative strategy in tackling the worldwide issue of plant diseases. The research highlights the crucial requirement for sophisticated computer vision technologies to guarantee food security and develop sustainable agriculture. Combining YOLOv4 and photo retrieval allows for precise and quick identification of plant diseases. This technology provides a reliable and effective way to identify new leaf diseases with no training required. This discovery places the study at the forefront of the confluence between deep learning and plant pathology, making major contributions to generating strong and adaptable solutions for the issues in modern agriculture. Continual improvements and fine-tuning of procedures are being made to promote the role of technology in protecting global food production and supporting agricultural sustainability in the future.

## Data availability statement

The original contributions presented in the study are included in the article/**Supplementary Material**. Further inquiries can be directed to the corresponding author.

## Author contributions

MZ: Conceptualization, Data curation, Formal analysis, Investigation, Methodology, Writing – original draft. EA: Funding acquisition, Methodology, Project administration, Resources, Writing – review & editing. AA: Conceptualization, Supervision, Validation, Visualization, Writing – review & editing.

## References

[B1] Albattah W. Nawaz M. Javed A. Masood M. Albahli S. (2022). A novel deep learning method for detection and classification of plant diseases. Complex Intelligent Syst., 1–18. doi: 10.1007/s40747-021-00536-1

[B2] Arathi B. Dulhare U. N. (2023). “Classification of cotton leaf diseases using transfer learning-denseNet-121,” in Proceedings of Third International Conference on Advances in Computer Engineering and Communication Systems: ICACECS 2022. (Hyderabad, India) 393–405.

[B3] Attallah O. (2023). Tomato leaf disease classification via compact convolutional neural networks with transfer learning and feature selection. Horticulture 9, 149. doi: 10.3390/horticulturae9020149

[B4] Bin Naeem A. Senapati B. Chauhan A. S. Kumar S. Gavilan J. C. O. Abdel-Rehim W. M. F. (2023). Deep learning models for cotton leaf disease detection with VGG-16. Int. J. Intelligent Syst. Appl. Eng. 11, 550–556.

[B5] Chen Y. Wu Q. (2023). Grape leaf disease identification with sparse data via generative adversarial networks and convolutional neural networks. Precis Agric. 24, 235–253. doi: 10.1007/s11119-022-09941-z

[B6] Chen Z. Ruhui W. Yiyan L. Chuyu L. Siyu C. Zhineng Y.. (2022). Plant disease recognition model based on improved YOLOv5. Agronomy 12, 365. doi: 10.3390/agronomy12020365

[B7] Chuanlei Z. Shanwen Z. Jucheng Y. Yancui S. Jia C. (2017). Apple leaf disease identification using genetic algorithm and correlation-based feature selection method. Int. J. Agric. Biol. Eng. 10, 74–83.

[B8] Dhinesh E. Jagan A.. (2019). “Detection of leaf disease using principal component analysis and linear support vector machine,” in 2019 11th International Conference on Advanced Computing (ICoAC). (Chennai, India) 350–355.

[B9] Eunice J. Popescu D. E. Chowdary M. K. Hemanth J. (2022). Deep learning-based leaf disease detection in crops using images for agricultural applications. Agronomy 12, 2395.

[B10] Hassan S. M. Maji A. K. Jasiński M. Leonowicz Z. Jasińska E. (2021). Identification of plant-leaf diseases using CNN and transfer-learning approach. Electron. (Basel) 10, 1388. doi: 10.3390/electronics10121388

[B11] Jasim M. A. Al-Tuwaijari J. M. (2020). “Plant leaf diseases detection and classification using image processing and deep learning techniques,” in 2020 International Conference on Computer Science and Software Engineering (CSASE). (Duhok, Iraq) 259–265.

[B12] Javidan S. M. Banakar A. Vakilian K. A. Ampatzidis Y. (2023). Diagnosis of grape leaf diseases using automatic K-means clustering and machine learning. Smart Agric. Technol. 3, 100081. doi: 10.1016/j.atech.2022.100081

[B13] Kaur P. Shilpi H. Rajeev T. Shuchi U. Surbhi B. Arwa M.. (2022). Recognition of leaf disease using the hybrid convolutional neural network by applying feature reduction. Sensors 22, 575. doi: 10.3390/s22020575 35062534 PMC8779777

[B14] Liu G. Peng J. El-Latif A. A. A. (2023). SK-mobileNet: A lightweight adaptive network based on complex deep transfer learning for plant disease recognition. Arab J. Sci. Eng. 48, 1661–1675. doi: 10.1007/s13369-022-06987-z

[B15] Liu J. Wang X. (2021). Plant diseases and pests detection based on deep learning: a review. Plant Methods 17, 1–18. doi: 10.1186/s13007-021-00722-9 33627131 PMC7903739

[B16] Mitra D. (2021). Emerging plant diseases: research status and challenges. Emerging Trends Plant Pathol. 1–17.

[B17] Mustafa M. S. Husin Z. Tan W. K. Mavi M. F. Farook R. S. M. (2020). Development of an automated hybrid intelligent system for herbs plant classification and early herbs plant disease detection. Neural Comput. Appl. 32, 11419–11441. doi: 10.1007/s00521-019-04634-7

[B18] Peng Y. Wang Y. (2022). Leaf disease image retrieval with object detection and deep metric learning. Front. Plant Sci. 13, 963302. doi: 10.3389/fpls.2022.963302 36176678 PMC9513793

[B19] Perveen K. Sanjay K. Sahil K. Mukesh S. Najla A. A. Shanzeh B.. (2023). Multidimensional attention-based CNN model for identifying apple leaf disease. J. Food Qual 2023, 1–12. doi: 10.1155/2023/9504186

[B20] Rahman S. U. Alam F. Ahmad N. Arshad S. (2023). Image processing based system for the detection, identification and treatment of tomato leaf diseases. Multimed Tools Appl. 82, 9431–9445. doi: 10.1007/s11042-022-13715-0

[B21] Roy A. M. Bhaduri J. (2021). A deep learning enabled multi-class plant disease detection model based on computer vision. Ai 2, 413–428. doi: 10.3390/ai2030026

[B22] Sangeetha K. Rima P. Kumar P. Preethees S. (2022). “Apple leaf disease detection using deep learning,” in 2022 6th International Conference on Computing Methodologies and Communication (ICCMC). (Erode, India) 1063–1067.

[B23] Sanida M. V. Sanida T. Sideris A. Dasygenis M. (2023). An efficient hybrid CNN classification model for tomato crop disease. Technol. (Basel) 11, 10. doi: 10.3390/technologies11010010

[B24] Sharma R. P. Dharavath R. Edla D. R. (2023). IoFT-FIS: Internet of farm things based prediction for crop pest infestation using optimized fuzzy inference system. Internet Things 21, 100658. doi: 10.1007/978-3-031-33808-3

[B25] Singh M. K. Chetia S. Singh M. (2017). Detection and classification of plant leaf diseases in image processing using MATLAB. Int. J. Life Sci. Res. 5, 120–124.

[B26] Soeb M. J. A. Fahad J. Tahmina A. Muhammad R. Fahim M. Aney P.. (2023). Tea leaf disease detection and identification based on YOLOv7 (YOLO-T). Sci. Rep. 13, 6078, 2023. doi: 10.1038/s41598-023-33270-4 37055480 PMC10102080

[B27] Taheri-Garavand A. Nasiri A. Fanourakis D. Fatahi S. Omid M. Nikoloudakis N. (2021). Automated in situ seed variety identification via deep learning: a case study in chickpea. Plants 10, 1406. doi: 10.3390/plants10071406 34371609 PMC8309301

[B28] Terentev A. Vladimir B. Ekaterina S. Dmitriy E. Danila E. Dmitriy K.. (2023). Hyperspectral remote sensing for early detection of wheat leaf rust caused by puccinia triticina. Agriculture 13, 1186. doi: 10.3390/agriculture13061186

[B29] Thangavel K. D. Seerengasamy U. Palaniappan S. Sekar R. (2023). Prediction of factors for Controlling of Green House Farming with Fuzzy based multi-class Support Vector Machine. Alexandria Eng. J. 62, 279–289.

[B30] Vengaiah C. Konda S. R. (2023). Improving tomato leaf disease detection with denseNet-121 architecture. Int. J. Intelligent Syst. Appl. Eng. 11, 442–448.

[B31] Wang J. Yu L. Yang J. Dong H. (2021). Dba_ssd: A novel end-to-end object detection algorithm applied to plant disease detection. Information 12, 474. doi: 10.3390/info12110474

[B32] Xinming W. Tang S. (2023). Comparative study on Leaf disease identification using Yolo v4 and Yolo v7 algorithm. AgBioForum, 25, 1.

[B33] Xu L. Cao B. Ning S. Zhang W. Zhao F. (2023). Peanut leaf disease identification with deep learning algorithms. Mol. Breed. 43, 25. doi: 10.1007/s11032-023-01370-8 37313521 PMC10248705

