# Supplementary material for: Detection and identification of plant leaf diseases using YOLOv4
Source: Front Plant Sci. 2024 Apr 22;15:1355941. doi: 10.3389/fpls.2024.1355941 (PMC11070553; doi:10.3389/fpls.2024.1355941)
Supplement: Supplementary file 1 [file DataSheet_1.docx]

**Supplementary file.**

Supplementary Table 1: Training Images Path Text File.

| ! Unzip / mydrive/yolov4/obj.zip -d data/ | | | | | |
| --- | --- | --- | --- | --- | --- |
| inflating : | data / yolov4_dataset / Potato_ | _healthy_a3ec91aa - 8503-4711 - a912-0d1427e25686 |  | RS | 5409_new90degFlipLR_annotated.jpg |
| inflating : | data / yolov4_dataset / | Potato__healthy_750584fd - 72e0-45c8 - b37c - 97208bd559cc |  | RS | 1885_newPixel25_annotated.jpg |
| inflating : | data / yolov4_dataset / | Potato__healthy_b35b9f8b - daca - 4d12 - a5be - defe68d0acc5_ | defe68d0acc5_ | RS | 1903 180deg_annotated.jpg |
| inflating : | data / yolov4_dataset / | Potato__healthy_8583c4ff - 2d8b - 4b6f - 89a8 - fcc98917ce9b |  | RS | 1961_270deg_annotated.jpg |
| inflating : | data / yolov4_dataset / Potato_ | _healthy_af15ac00-67ff - 4114 - ace8-6dca48b94665 |  | RS | 1865_flipTB_annotated.jpg |
| inflating : | data / yolov4_dataset / Potato_ | healthy_929107f1-4ae9-4dae - 8319 - ae7cf4804236 |  | RS | 1932_annotated.jpg |
| inflating : | data / yolov4_dataset / Potato_ | healthy_alabff90-34e5-464d - b4d3-0c09f5924900 |  | RS | 5414_flipLR_annotated.jpg |
| inflating : | data / yolov4_dataset / Potato | ____ healthy_5e19b1f4 - fd65-4c4d - bcd2-18c8c519eddb |  | RS | 1965_new30degFlipTB_annotated.jpg |
| inflating : | data / yolov4_dataset / Potato | ____ healthy_57e24d88 - fd01-4422 - b8b5 - d688d2296184 |  | RS | 5401_270deg_annotated.jpg |
| inflating : | data / yolov4_dataset / | Potato__healthy_923d4cc3-021a - 4bcd - 9f5f - | 1d6f69e4ad33_ | RS | 1888_new200degFlip LR_annotated.jpg |
| inflating : | data / yolov4_dataset / | Potato__healthy_5a3fc4bb - a78c - 4276-8934 - febe37ffb860 |  | RS | 5412_new200degFlipLR_annotated.jpg |
| inflating : | data / yolov4_dataset / | Potato__healthy_5d505af8-3f73-4781-980c - a4a696a04550 |  | RS | 1934_flipTB_annotated.jpg |
| inflating : | data / yolov4_dataset / Potato | healthy_799b10e8 - ba67-4e08-9abe - 748789572ad1 |  | RS | 1881_newGRR_annotated.jpg |
| inflating : | data / yolov4_dataset / Potato_ | healthy_4ae82355-6885-40e7-9807 - dabe46ed3441 |  | RS | 5410_new30degFlipTB_annotated.jpg |
| inflating : | data / yolov4_dataset / Potato_ | _healthy_a02b6205 - b40a - 4178-8b34-3494f84b9d1f |  | RS | 1958_newGGR_annotated.jpg |
| inflating : | data / yolov4_dataset / Potato_ | healthy_683b04ad - 7941-4819-9965 - ba32c725eb22 |  | RS | 1861_newGGR_annotated.jpg |
| inflating : | data / yolov4_dataset / Potato_ | _healthy_ad9b6cf4 - e065-496d - 91c7-5713ce14e19e |  | RS | 1926_180deg_annotated.jpg |
| inflating : | data / yolov4_dataset / Potato_ | healthy_5fcbde8f - 52af - 4963 - b324 - ff7f4dd6bd4c |  | RS | 1762_flipTB_annotated.jpg |
| inflating : | data / yolov4_dataset / Potato_ | _healthy_9b5be0c5-7d7e - 493d - 88ba - | c721c85f73ca_ | RS | 5405_new90degFlipTB_annotated.jpg |
| inflating | data / volov4 dataset / Potato | healthy Ahhrrfh6-572A - 4r80-9h37 - Ar3ed8000rof |  | RS | 1701 18Adeg annotated ing |


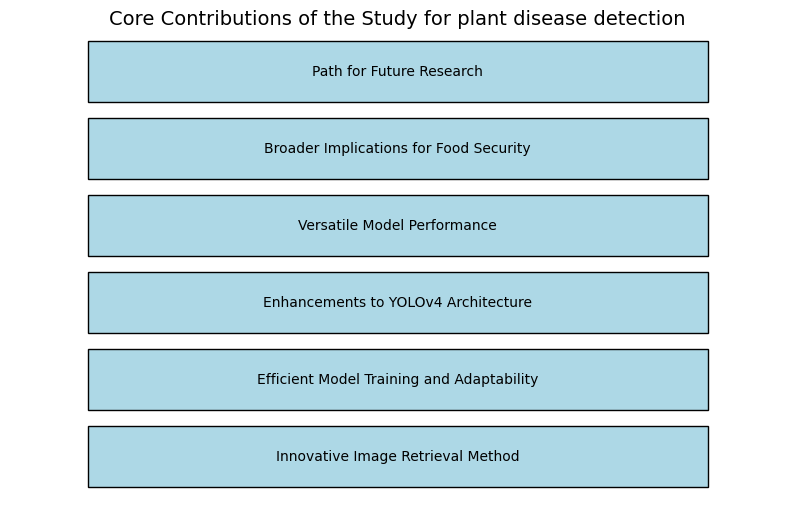


Supplementary Figure 1: Core Contributions of study.


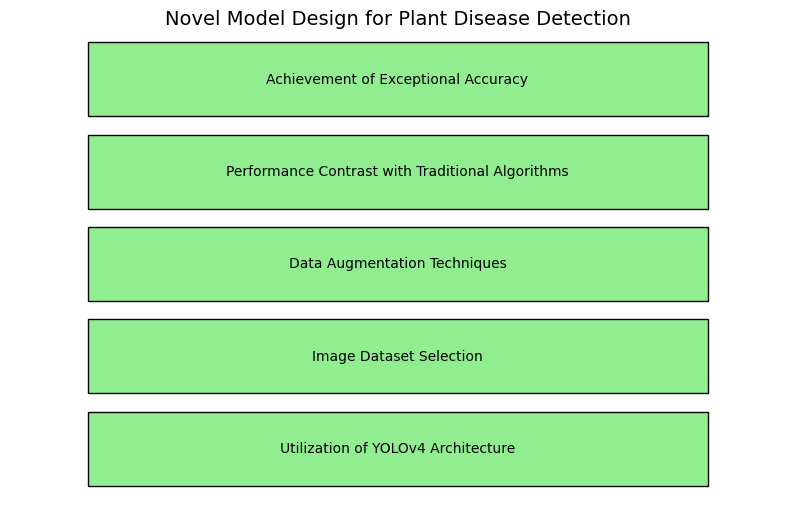


Supplementary Figure 2: Model Novel Design Approach for Plant Disease Detection.


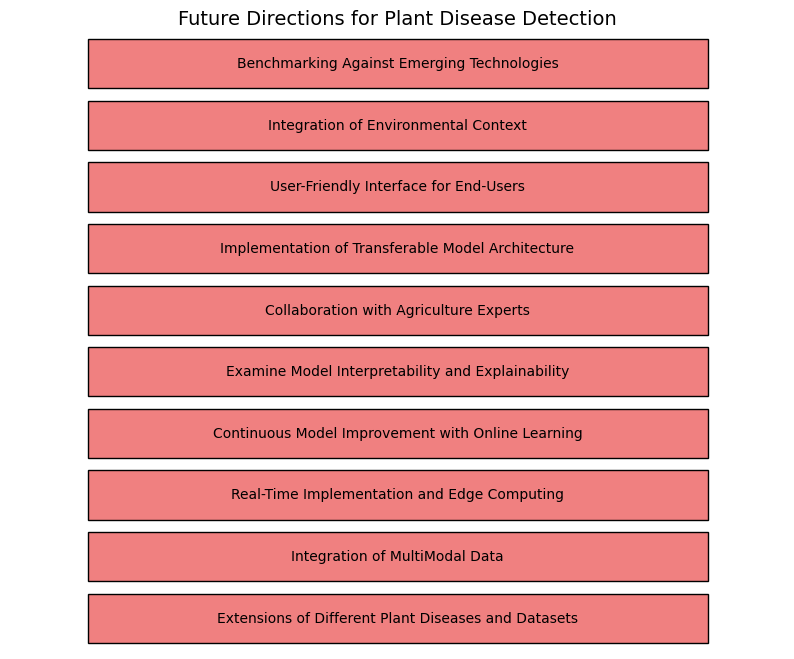


Supplementary Figure 3: Future directions for plant disease detection.
